# The HDAC6/APOBEC3G complex regulates HIV-1 infectiveness by inducing Vif autophagic degradation

**DOI:** 10.1186/s12977-015-0181-5

**Published:** 2015-06-24

**Authors:** María-Soledad Valera, Laura de Armas-Rillo, Jonathan Barroso-González, Serena Ziglio, Julien Batisse, Noé Dubois, Sara Marrero-Hernández, Sophie Borel, Laura García-Expósito, Martine Biard-Piechaczyk, Jean-Christophe Paillart, Agustín Valenzuela-Fernández

**Affiliations:** Laboratorio de Inmunología Celular y Viral, Unidad de Farmacología, Departamento de Medicina Física y Farmacología, Facultad de Medicina, Universidad de La Laguna (ULL), Campus de Ofra s/n, 38071 La Laguna, Tenerife Spain; Architecture et Réactivité de l’ARN, CNRS, Institut de Biologie Moléculaire et Cellulaire, Université de Strasbourg, 15 rue René Descartes, 67084 Strasbourg, France; Centre d’études d’agents Pathogènes et Biotechnologies pour la Santé (CPBS) UMR5236 CNRS UMSF, 1919 route de Mende, 34293 Montpellier Cedex 5, France

**Keywords:** HIV-1, HDAC6, APOBEC3G, Vif, CBF-β, Anti-HIV-1 restriction complex, Autophagic clearance

## Abstract

**Background:**

Human immunodeficiency virus type 1 (HIV-1) has evolved a complex strategy to overcome the immune barriers it encounters throughout an organism thanks to its viral infectivity factor (Vif), a key protein for HIV-1 infectivity and in vivo pathogenesis. Vif interacts with and promotes “apolipoprotein B mRNA-editing enzyme-catalytic, polypeptide-like 3G” (A3G) ubiquitination and subsequent degradation by the proteasome, thus eluding A3G restriction activity against HIV-1.

**Results:**

We found that cellular histone deacetylase 6 (HDAC6) directly interacts with A3G through its C-terminal BUZ domain (residues 841–1,215) to undergo a cellular co-distribution along microtubules and cytoplasm. The HDAC6/A3G complex occurs in the absence or presence of Vif, competes for Vif-mediated A3G degradation, and accounts for A3G steady-state expression level. In fact, HDAC6 directly interacts with and promotes Vif autophagic clearance, thanks to its C-terminal BUZ domain, a process requiring the deacetylase activity of HDAC6. HDAC6 degrades Vif without affecting the core binding factor β (CBF-β), a Vif-associated partner reported to be key for Vif- mediated A3G degradation. Thus HDAC6 antagonizes the proviral activity of Vif/CBF-β-associated complex by targeting Vif and stabilizing A3G. Finally, in cells producing virions, we observed a clear-cut correlation between the ability of HDAC6 to degrade Vif and to restore A3G expression, suggesting that HDAC6 controls the amount of Vif incorporated into nascent virions and the ability of HIV-1 particles of being infectious. This effect seems independent on the presence of A3G inside virions and on viral tropism.

**Conclusions:**

Our study identifies for the first time a new cellular complex, HDAC6/A3G, involved in the autophagic degradation of Vif, and suggests that HDAC6 represents a new antiviral factor capable of controlling HIV-1 infectiveness by counteracting Vif and its functions.

**Electronic supplementary material:**

The online version of this article (doi:10.1186/s12977-015-0181-5) contains supplementary material, which is available to authorized users.

## Background

APOBEC3G (apolipoprotein B mRNA-editing enzyme-catalytic, polypeptide-like 3G or A3G) is a member of the APOBEC superfamily of cytidine deaminases [1], which are thought to restrict human immunodeficiency virus type 1 (HIV-1) activity. Following its incorporation into nascent virions, A3G exerts its antiviral effects by deaminating cytidine to uracil in newly synthesized (−)-strand viral DNA, leading to G-to-A invalidating hypermutations in the (+)-strand viral DNA [2–5]. The DNA repair pathway involves uracil-DNA glycosylase while apurinic–apyrimidinic endonuclease degrades A3G-hypermutated viral DNA [6–8]. In contrast, non-degraded hypermutated (+)-strand viral DNA potentially leads not only to mutated viral proteins and/or altered reading frames, but also to unexpected translation termination codons [2, 3, 5, 9–12]. Additionally, A3G also inhibits HIV-1 infection by a cytidine deaminase-independent pathway involving interaction with viral RNA and impairment of the reverse transcription and integration processes [4, 8, 11, 13–17]. Thus, the ability of A3G to interact with different cellular and/or viral RNA and proteins could compromise or promote its anti-HIV-1 functions [18–20]. A3G might, however, diversify the viral genome, thereby promoting infectious HIV-1 variants and even generating HIV-1 drug-resistant phenotypes [21–24]. These data suggest the existence of a complex A3G-mediated restrictive function against HIV-1, potentially dependent on a variety of A3G targets [12, 25, 26].

HIV-1 employs a complex strategy to overcome immune barriers [27, 28], particularly in target cells, through its auxiliary viral genes, such as the viral infectivity factor (Vif), which is essential for HIV-1 infectivity and in vivo pathogenesis [28–30]. In fact, Vif promotes A3G degradation, eluding its restrictive activity against HIV-1 [31, 32]. Vif binds to several cellular proteins, such as Cullin 5 (Cul5), Elongin B (EloB), EloC, and Rbx2 to form a Vif-BC-Cul5 complex, acting as an ubiquitin ligase (E3)-like complex [32–36]. This Vif-BC-Cul5 complex targets A3G, via Vif-A3G interactions, thus mediating A3G ubiquitination and subsequent proteasome degradation [32–35]. Vif also requires and interacts with the core binding factor β (CBF-β), the non-DNA-binding subunit of a heterodimeric transcription factor, to degrade A3G [36–38]. By this mechanism, Vif indirectly protects viral DNA from A3G deaminations and avoids A3G incorporation into nascent viral particles [32, 39–41]. Moreover, Vif also negatively controls A3G antiviral activity by targeting its mRNA and inhibiting its translation [40, 42, 43].

We have previously reported that HIV-1 stabilizes acetylated microtubules (MT) during early infection, a crucial signal for HIV-1 Env-mediated pore fusion formation, viral entry, and infection [44, 45]. Human histone deacetylase 6 (HDAC6) not only inhibits this early HIV-1 Env/CD4-mediated signalling, but also viral infection and replication, in a deacetylase-dependent manner [44–46]. Furthermore, HDAC6 promotes the formation of aggresomes and activates autophagy [47–51]. Hence, HDAC6 is involved in the transport and clearance of those ubiquitinated proteins that do not enter the proteasome pathway, and that bear unconjugated C-terminal diglycine motifs on their ubiquitin-associated chains [47–52]. In cells where proteasomal clearance is blocked, HDAC6 may also recognize unanchored, protein-free polyubiquitin chains. This event activates HDAC6-triggered actinomyosin- and autophagy-dependent aggresome processing of proteins [53]. Thus, HDAC6 emerges as a key enzyme for clearing ubiquitinated proteins that fail to enter proteasome pathways. In fact, HDAC6 presents a Cys/His-rich motif in its C-terminal region [54–56], which interacts in particular with ubiquitin and could function as a mono-ubiquitin ligase [51, 52, 57–59]. This motif shows significant sequence homology with BRCA1-associated protein BRAP2 and several ubiquitin-specific proteases, and is known as a DAUP (deacetylase-ubiquitin-specific protease) domain [60], HUB (HDAC6-, USP3- and BRAP2-related) finger [61], ZnF-UBP (ubiquitin C-terminal hydrolase-like zinc finger) [57], PAZ (polyubiquitin-associated zinc finger) [58], or BUZ (bound to ubiquitin zinc finger) domain [47]. Of note, Vif seems to be post-translationally mono-ubiquitinated, being stable and not recruited by the proteasome-degradative pathway [62, 63].

Considering the anti-HIV-1 and pro-aggresome/autophagic functions of HDAC6, it is conceivable that HDAC6 regulates HIV-1 infection by the interplay of A3G and Vif proteins, and the Vif-triggered ubiquitination and subsequent proteasome degradation of A3G. In this study, we show that HDAC6 forms a constitutive complex with A3G that competes for Vif-A3G interaction. This new complex accounts for the steady-state expression level of A3G, thereby neutralizing Vif-mediated A3G ubiquitination and proteasome degradation, and stabilizing Vif-non-targeted A3G. HDAC6 is able to establish a ternary complex through binding to A3G and Vif at the same time. In fact, HDAC6 interacts with Vif and regulates its stability without altering CBF-β. Finally, HDAC6 controls HIV-1 infectiveness by regulating the incorporation of Vif into nascent virions through the induction of its autophagic degradation. This mechanism is dependent on the BUZ domain of HDAC6 and requires its deacetylase activity.

Altogether, our results pointed out HDAC6 as a new protein involved in the antiviral machinery of an HIV-1 infected cell.

## Results

### HDAC6 induces Vif degradation, and protects A3G from Vif-mediated degradative activity

**Figure 1:**
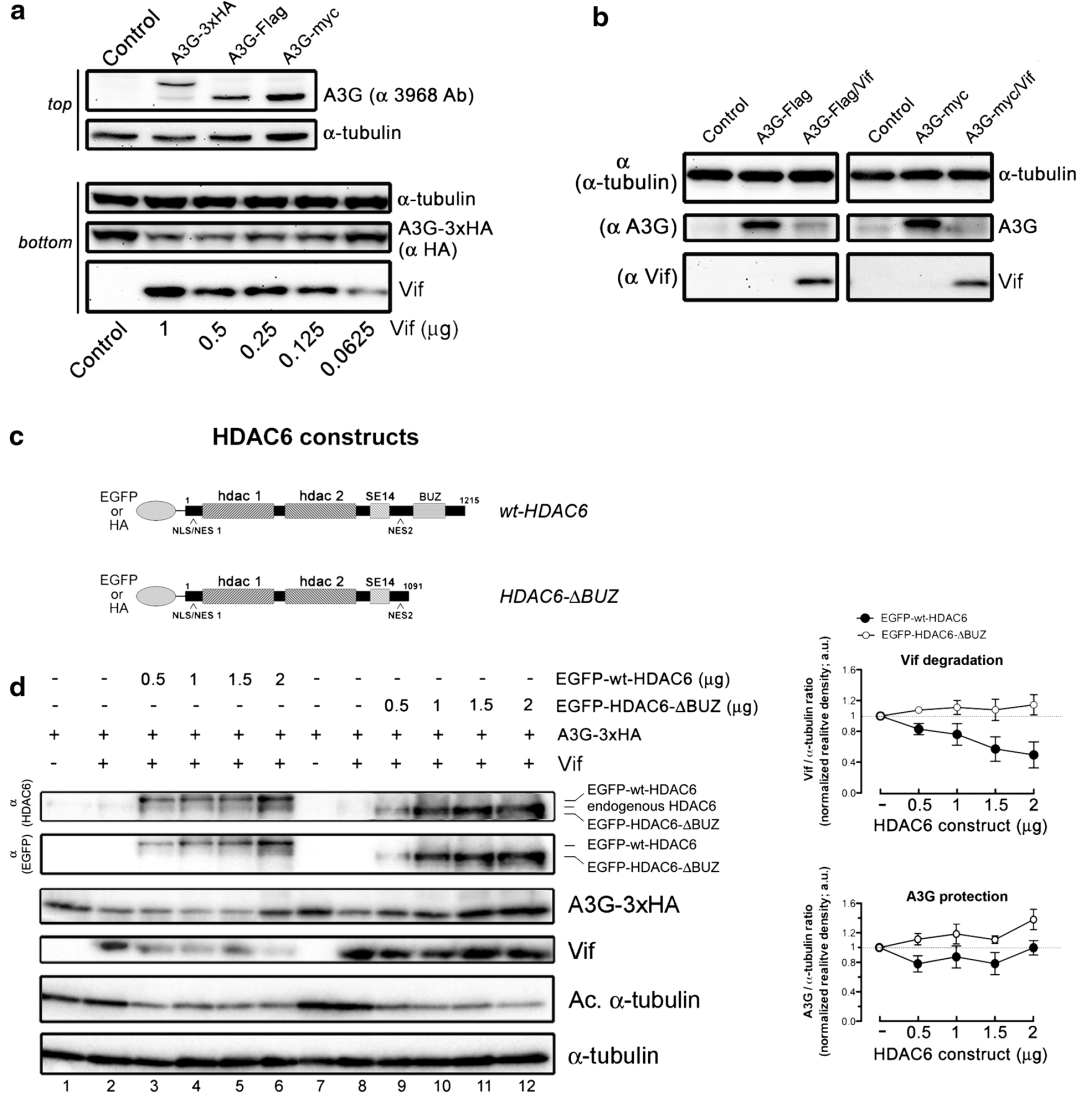
HDAC6 degrades Vif and protects A3G from Vif-mediated degradation. **a**, **b** *Top*-*A*, western blot analysis of different A3G-tagged proteins in cells lacking endogenous A3G expression (control). *Bottom* **a**, **b**, western blot analysis of Vif-mediated degradation of different A3G-tagged molecules. **c** Schematic representation of EGFP- or HA-tagged wt-HDAC6 and HDAC6-ΔBUZ constructs used in this study. The different domains are indicated: the nuclear localization signal (NLS) and export signal 1 and 2 (NES1/2), the two histone deacetylase domains (hdac 1 and 2) together with the SE14 region, and the BUZ domain (adapted from [56]). **d** *Left*, western blot of dose-response effects of EGFP-wt-HDAC6 and EGFP-HDAC6-ΔBUZ (detected by anti-HDAC6 or anti-EGFP abs, respectively) on Vif and A3G-3xHA. *Lanes* *1*, *7* and *2*, *8* are controls representing cells overexpressing only A3G-3xHA or A3G-3xHA together with Vif, respectively. *Right*, quantitation of western blots regarding EGFP-HDAC6- and EGFP-HDAC6-ΔBUZ-mediated Vif degradation (*top*) and A3G protection (*bottom*). In all western blots, α-tubulin is the control for total protein. When indicated, acetylated α-tubulin is a read-out for functional deacetylase activity of the different HDAC6 construct. All experiments were performed in HEK 293T cells. When indicated, endogenous HDAC6 expression level is shown. Data are expressed as mean ± S.E.M. of six independent experiments.

To ascertain the ability of HDAC6 to protect A3G against Vif-mediated proteasome degradation, we analyzed A3G degradation by recombinant Vif in HEK 293T permissive cells (lacking A3G), under control conditions (Figure 1a, b) or with overexpressed HDAC6 constructs (Figure 1c). We observed that Vif degrades A3G in a dose-dependent manner, regardless of the A3G-associated tag (Figure 1a, b). However, in presence of overexpressed wt-HDAC6 (Figure 1d), we observed a dose-dependent protective effect against Vif-mediated A3G degradation, which correlated with an extent degradation of Vif (Figure 1d, quantitated in right panel). In contrast, the overexpression of HDAC6-ΔBUZ, lacking the C-terminal region that bears the Cys/His-rich BUZ motif [56], did not promote Vif degradation and strongly protected A3G from Vif degradation (Figure 1d, quantitated in right panel). This may indicate a potential interaction of HDAC6 with A3G, and/or an HDAC6-triggered decoupling of A3G from the proteasome-induced degradative action of Vif.

**Figure 2:**
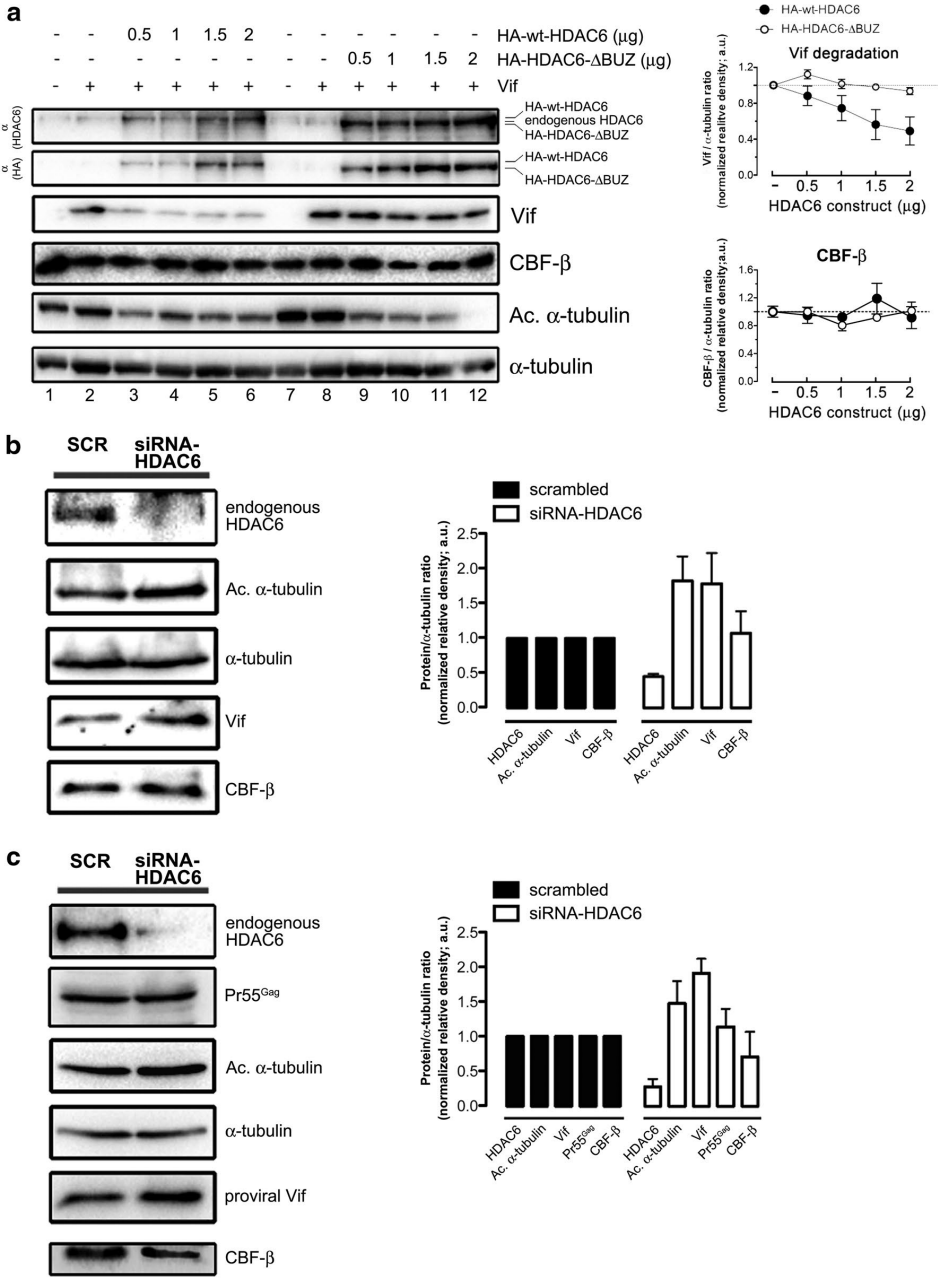
HDAC6 reduces Vif and endogenous CBF-β expression. **a** *Left*, western blot of dose–response effects of HA-wt-HDAC6 and HA-HDAC6-ΔBUZ (detected with anti-HDAC6 or anti-HA abs) on over-expressed Vif and endogenous CBF-β in the absence of A3G. *Lanes* *1*, *7* and *2*, *8* are controls without or with overexpressing Vif, respectively. *Right*, quantitation of western blots regarding HA-HDAC6- and EGFP-HDAC6-ΔBUZ-mediated Vif degradation. **b**, **c** Cell lysates from control scrambled (SCR) or siRNA-HDAC6-treated cells, expressing recombinant or proviral Vif (pNL4.3-HIV-1). The level of expression of Vif, proviral Pr55^Gag^ when indicated and endogenous CBF-β are shown in each condition. *Right*, histogram indicates the level of expression of each protein analyzed and normalized by the amount of total α-tubulin, under any experimental condition. In all western blots, α-tubulin is the control for total protein. Acetylated α-tubulin is a read-out for functional deacetylase activity of the remaining endogenous HDAC6. Endogenous HDAC6 expression level is shown. All experiments were performed in HEK 293T cells. Data are expressed as mean ± S.E.M. of six independent experiments.

Interestingly, in the absence of A3G, we also observed that HDAC6 reduced Vif expression in a BUZ domain- and dose-dependent manner (Figure 2a, quantitated in right panel).

In cells, Vif recruits the transcription factor CBF-β together with EloC/B, Cul5 and Rbx to form an E3-ubiquitin ligase complex. In fact, CBF-β stabilizes Vif folding and its proviral function in the Vif-Cul5 E3 ligase complex [37, 38, 64]. In order to analyze the effect of HDAC6 on the stability of CBF-β in the absence of A3G, we over-expressed Vif in HEK 293T cells and showed that wt-HDAC6 degrades Vif in a dose- and BUZ-dependent manner, without affecting endogenous CBF-β (Figure 2a, quantitated in right panel). Interestingly, specific knock-down of endogenous HDAC6 by small interfering RNA (siRNA) enhanced the expression level of recombinant (Figure 2b) or proviral (Figure 2c) Vif without altering the expression of endogenous CBF-β. In parallel, we observed an increase on acetylated α-tubulin level (Figure 2b, c, quantitated in right panel). These results suggest that the endogenous HDAC6 acts on Vif modulating its expression level. Indeed, HDAC6 may also protect A3G by targeting the Vif-Cul5 E3 ligase/CBF-β complex by degrading Vif, which nucleates this complex.

**Figure 3:**
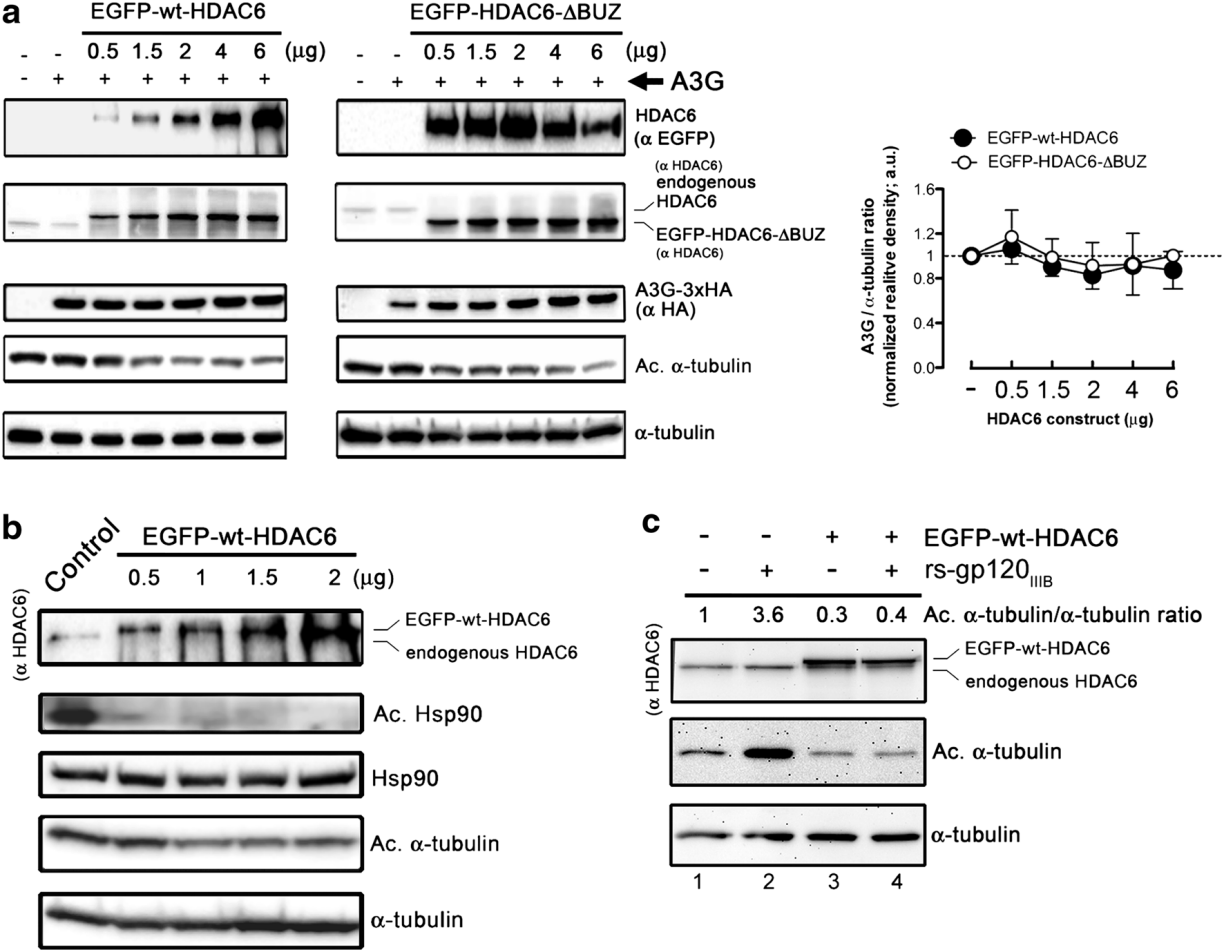
Effect of HDAC6 on A3G expression and substrate deacetylation. **a** *Left*, western blot of dose–response effects of EGFP-wt-HDAC6 and EGFP-HDAC6-ΔBUZ (detected by anti-HDAC6 or anti-EGFP abs) on A3G-3xHA expression. *Right*, quantitation of western blots regarding EGFP-HDAC6- and EGFP-HDAC6-ΔBUZ-mediated effect on A3G expression. All experiments were performed in HEK 293T cells. When indicated, endogenous HDAC6 expression level is shown. All western blots are representative of three independent experiments. **b** Western blot of EGFP-wt-HDAC6-mediated Hsp90 deacetylation in HEK 293T cells in a dose–response assay. Acetylated (Ac.) Hsp90 was detected from endogenous Hsp90 pulled-down from cell lysates and with an anti-Hsp90 Ab, then blotted with a specific anti-acetylated Lys Ab. **c** Western blot of EGFP-wt-HDAC6-mediated α-tubulin deacetylation (compare *lanes* *1* and *3*) and inhibition of HIV-1 Env (rs-gp120_IIIB_)-induced acetylation of α-tubulin (compare *lanes* *2* and *4*), in a CD4 + T CEM.NKR-CCR5 cell line. In **b**, **c**, EGFP-wt-HDAC6 and acetylated (Ac.) α-tubulin were detected by anti-EGFP and anti-acetylated α-tubulin Abs, respectively. From **a** to **c**, α-tubulin is the control for total protein. When indicated, endogenous HDAC6 expression level is shown. All western blots are representative of three independent experiments.

In the absence of Vif, A3G was not degraded by the overexpression of wt-HDAC6 or HDAC6-ΔBUZ constructs (Figure 3a). The EGFP-wt-HDAC6 construct used herein is functional, since it deacetylates two of its main substrates, Hsp90 and acetylated α-tubulin (Figure 3b), and inhibits HIV-1 Env-gp120-mediated α-tubulin acetylation in CD4 + T cells (Figure 3c), as previously described [44, 45].

Taken together, our results show for the first time that HDAC6, through its C-terminal BUZ domain, induces the degradation of Vif without affecting CBF-β and protects A3G from its Vif-induced degradation.

### HDAC6 interacts with A3G

**Figure 4:**
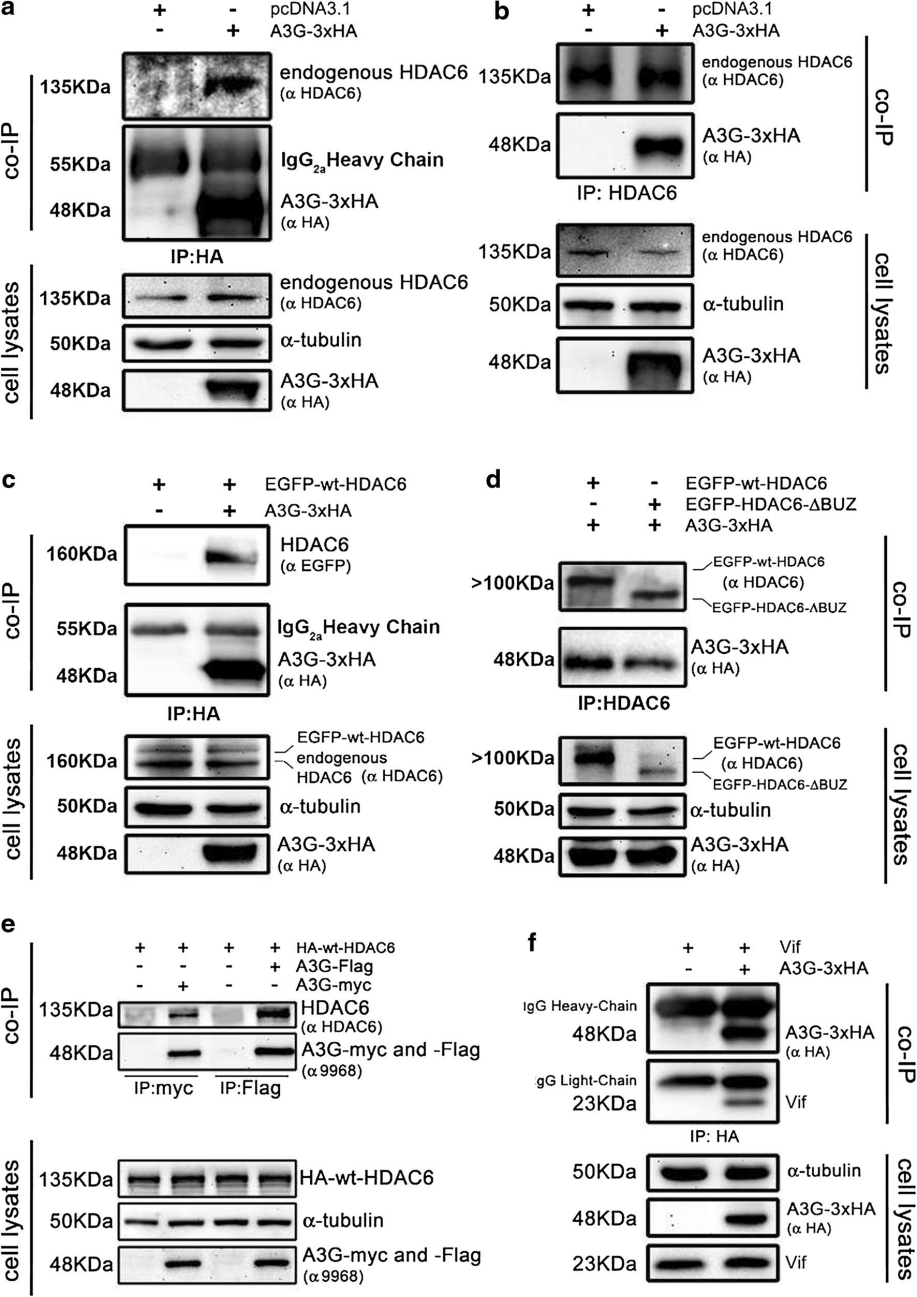
Co-immunoprecipitation of HDAC6 and A3G. **a** Cell lysates from control (empty pcDNA3.1 vector) or A3G-3xHA-expressing HEK 293T cells were subjected to co-immunoprecipitation (co-IP) with anti-HA Ab, followed by immunoblotting with HDAC6 and HA Abs. **b** Cell lysates from control [as in (**a**)] or A3G-3xHA-expressing cells were subjected to co-IP with anti-HDAC6 Ab, followed by immunoblotting with HDAC6 and HA Abs. **c** Cell-lysates from EGFP-wt-HDAC6-expressing or A3G-3xHA/EGFP-wt-HDAC6-co-expressing cells were subjected to co-IP with anti-HA Ab, followed by immunoblotting with EGFP and HA Abs. **d** Cell-lysates from A3G-3xHA/EGFP-wt-HDAC6- or A3G-3xHA/EGFP-HDAC6-ΔBUZ-co-expressing cells were subjected to co-IP with anti-HDAC6 Ab, followed by immunoblotting with EGFP and HA Abs. **e** Cell-lysates from A3G-Flag- or A3G-myc-expressing cells or cells co-expressing HA-wt-HDAC6 with each of the two A3G constructs were subjected to co-IP with anti-myc or anti-Flag Ab, followed by immunoblotting with HDAC6 or A3G Abs. **f** Cell-lysates from control cells (overexpressing recombinant Vif) or A3G-3xHA/Vif-co-expressing cells were subjected to co-IP with anti-HA Ab, followed by immunoblotting with Vif and HA Abs. From **a** to **f**, input expression levels of endogenous HDAC6 or overexpressed constructs are shown in cell lysates-western blots from HEK 293T cells. α-tubulin is the control for total protein. When detected, an IgG_2a_ heavy chain of IgG used to pull-down is indicated. Data are representative of three independent experiments.

We next sought to study whether HDAC6 interacts with A3G in the absence of Vif. By co-immunoprecipitation in HEK 293T cells (against A3G or endogenous HDAC6), we observed that A3G interacts with endogenous HDAC6 (Figure 4a, b). Similarly, we found that A3G co-immunoprecipitated with overexpressed EGFP-HDAC6 wild-type or ΔBUZ proteins (Figure 4c, d; against A3G or HDAC6, respectively). To ensure these results were not due to the tags used in A3G or HDAC6 constructs, we co-immunoprecipitated overexpressed HA-wt-HDAC6 with either A3G-myc or A3G-Flag proteins, thereby confirming the existence of these A3G/HDAC6 interactions (Figure 4e). As a control for specificity, we co-immunoprecipitated A3G-HA with Vif in HEK 293T cells and confirmed their binding (Figure 4f).

**Figure 5:**
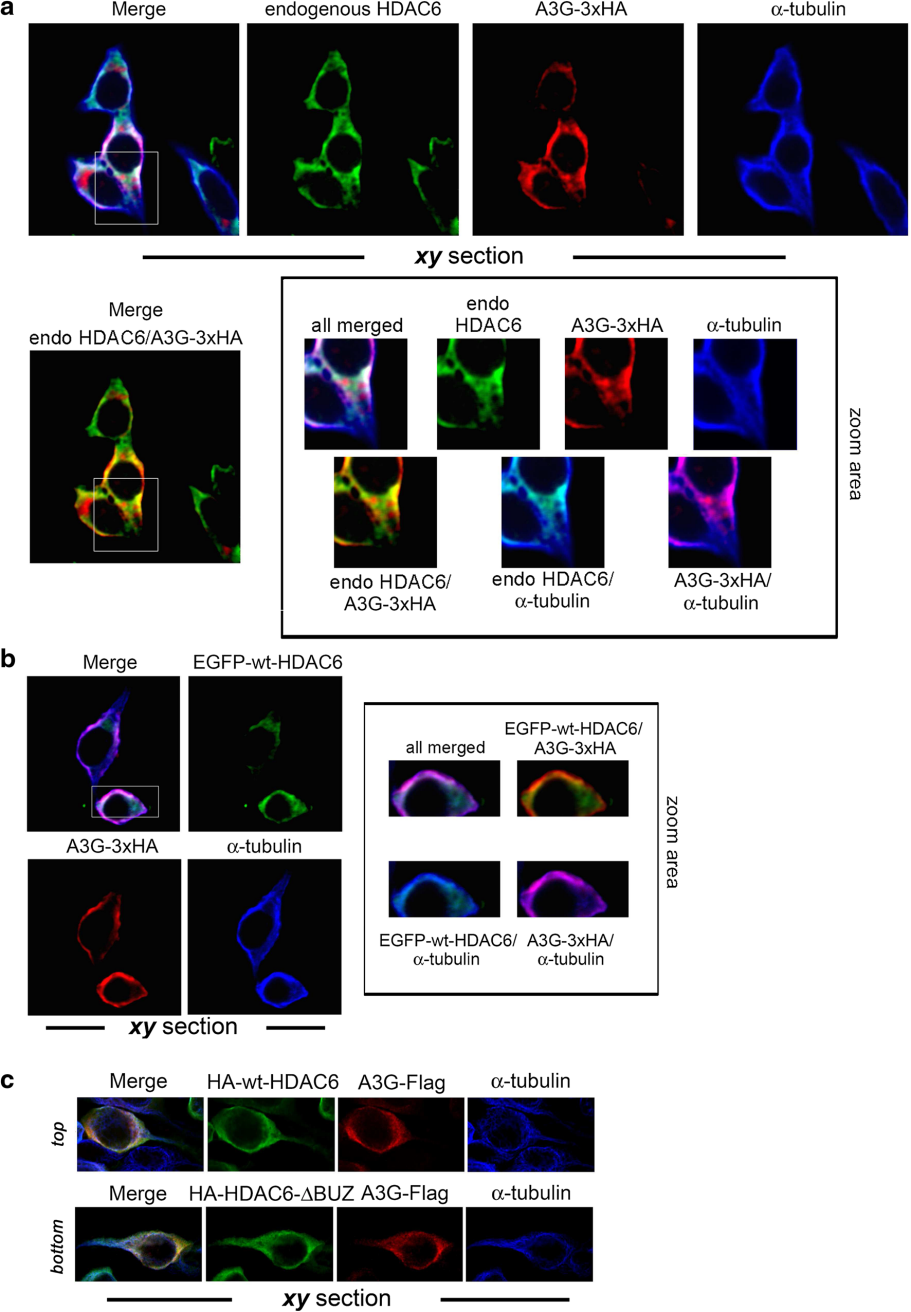
HDAC6 and A3G co-localize along microtubules in cells. **a** A series of confocal images, *xy* sections, shows expression patterns for endogenous HDAC6 and α-tubulin, and overexpressed A3G-3xHA. Different merged images are shown to display endogenous HDAC6/overexpressed A3G-3xHA co-distribution and co-distribution of this complex in the cytoplasm or upon MTs (α-tubulin). In the zoom area (*black-bordered inset*) is shown the expression of these proteins and their co-distribution, as indicated. **b** Series of confocal images, ***xy*** sections, shows expression pattern for overexpressed EGFP-wt-HDAC6 or A3G-3xHA and α-tubulin. Merged image is shown to display EGFP-wt-HDAC6/A3G-3xHA co-distribution in cytoplasm or upon MTs (α-tubulin). In the zoom area (*black-bordered inset*) is shown the expression of these proteins and their co-distribution, as indicated. **c** Series of confocal images, *xy* sections, shows expression pattern for overexpressed HA-wt-HDAC6, HA-HDAC6-ΔBUZ or A3G-Flag and α-tubulin. Merged image shows HA-wt-HDAC6/A3G-Flag (*top*) or HA-HDAC6-ΔBUZ/A3G-Flag (*bottom*) co-distribution in cytoplasm or upon MTs (α-tubulin). From **a** to **c**, data are representative of three independent experiments in HEK 293T cells.

Next, using fluorescence confocal microscopy, we observed that A3G co-distributed with endogenous HDAC6 or over-expressed HDAC6, either upon MT structures (Figure 5a, b; merged images and inset zoom area) where HDAC6 association occurs [44, 45, 65, 66], or in the cytoplasm (Figure 5c). This co-localisation was independent on the tag used (HA or Flag for A3G; EGFP and HA for HDAC6) (Figure 5b, c) and on the BUZ domain of HDAC6 (Figure 5c).

Taken together, this set of experiments indicates that HDAC6 interacts with A3G to form an HDAC6/A3G complex.

**Figure 6:**
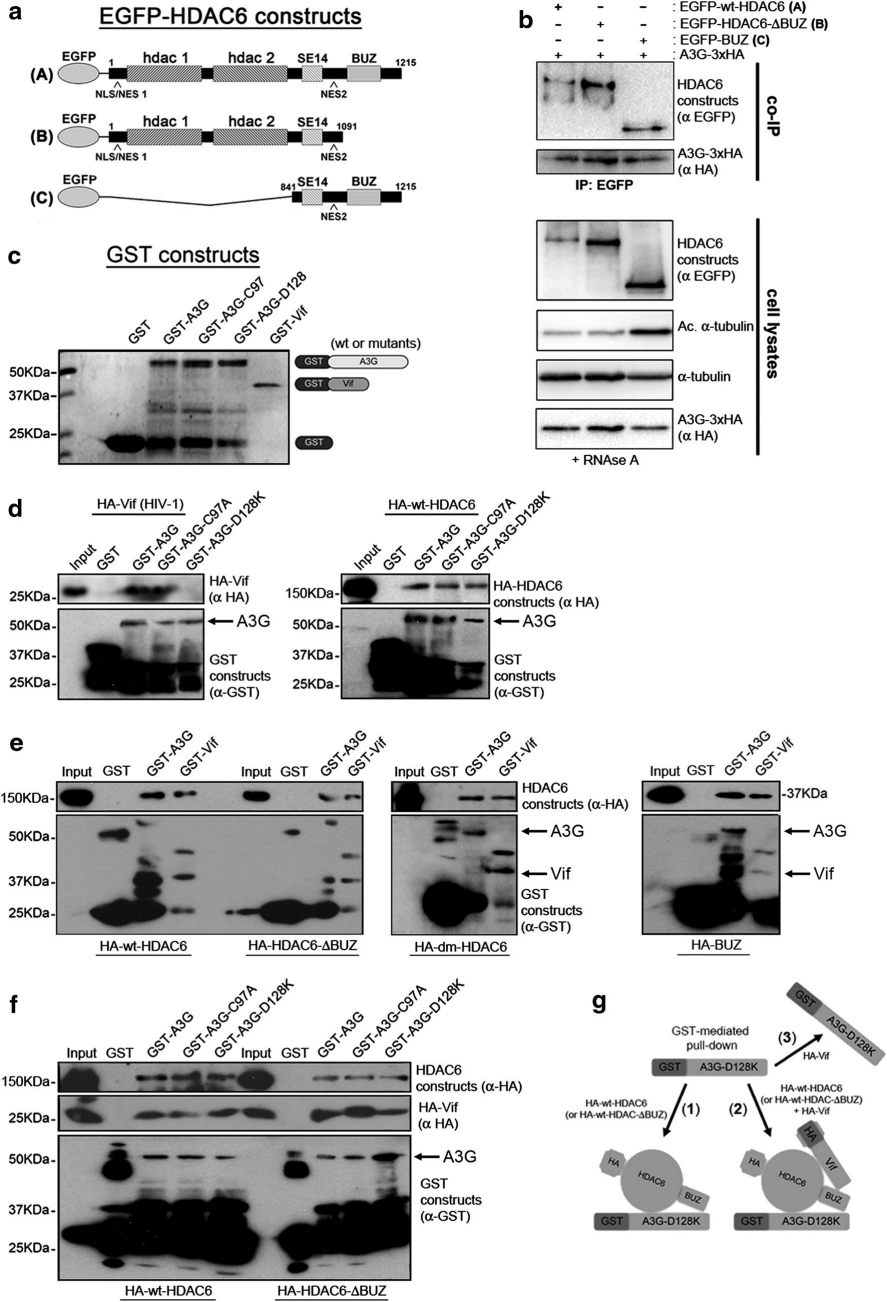
HDAC6 directly interacts with A3G and Vif to form a ternary complex. **a** Schematic representation of EGFP-HDAC6 constructs used in this assay. The constructs are (*A*) EGFP-wt-HDAC6, (*B*) a construct lacking the BUZ domain (EGFP-HDAC6-ΔBUZ) and (*C*) a construct bearing the BUZ domain. **b** RNAse A-treated cell lysates from HEK 293T cells co-expressing A3G-3xHA and each of the three EGFP-HDAC6 constructs [see (**a**)], were subjected to co-IP with anti-EGFP Ab, followed by immunoblotting with specific Abs against EGFP or HA. Input expression levels of each EGFP-HDAC6 construct used and overexpressed A3G-3xHA are shown in cell lysates-Western blots. α-tubulin was the control for total protein. Data shown are representative of three experiments. **c**–**f** Direct in vitro interaction of HDAC6 with A3G and Vif. *Coomassie blue staining* shows the electrophoretic migration profile of the recombinant GST, GST-A3G, GST-A3G mutants (C97A and D128K) and GST-Vif fusion proteins (**c**). Recombinant GST-proteins were incubated with purified in vitro translated HA-Vif [(**d**), *left panel*] or HA-HDAC6 proteins [(**e**); and (**d**), *right panel*] or both HA-Vif and HA-HDAC6 proteins (**f**). In **f**, a ternary GST-A3G-D128K/HA-HDAC6 construct-HA-Vif is shown. Bound Vif and HDAC6 proteins were fractionated on a 10% SDS-PAGE, followed by immunoblotting with anti-HA antibody (*upper panels*). Anti-GST immunoblot shows the loading of the assayed GST-fusion proteins (*lower panels*). From **b** to **f**, a representative experiment of three is shown. **g** A scheme showing GST-mediated A3G-D128K established interactions, as follows: (*1*) with HDAC6 (wt or ΔBUZ constructs); and (*2*) with HDAC6 (wt or ΔBUZ constructs) in the presence of Vif to form a ternary A3G-D128K/HDAC6-Vif complex. In (*3*) it is represented the inability of Vif to interact with A3G-D128K.

In order to ascertain the functional involvement of the C-terminal BUZ domain of HDAC6 in its interaction with A3G, we co-immunoprecipitated A3G against three different HDAC6 constructs: (A) EGFP-wt-HDAC6; (B) EGFP-HDAC6-ΔBUZ (lacking the BUZ domain); and (C) EGFP-HDAC6-BUZ (containing only the C-terminal Cys/His-rich BUZ motif) (Figure 6a) [56]. These co-immunoprecipitations were performed from HEK 293T cell lysates treated with RNAse A to avoid the possibility that protein–protein interactions were due to the ability of A3G to interact with nucleic acids or to a potential non-specific HDAC6-associated nucleic acid recruitment. A3G was independently co-immunoprecipitated with EGFP-wt-HDAC6, EGFP-HDAC6-ΔBUZ and EGFP-HDAC6-BUZ constructs (Figure 6b), suggesting the BUZ domain by itself is required, but not totally indispensable, for the HDAC6/A3G interaction. Thus, this could indicate that redundant A3G interacting motifs exist in the full-length HDAC6 protein.

### HDAC6 directly interacts with A3G and Vif, and forms an A3G/HDAC6-Vif complex

To further understand the involvement of HDAC6 in a binary (A3G/HDAC6; HDAC6-Vif) or ternary (A3G/HDAC6-Vif) complex, we analyzed protein interactions in vitro by GST pull-down assay (Figure 6c, GST constructs). First, we confirmed the interaction between A3G and Vif by pulling-down HA-Vif with GST-A3G (Figure 6d). As previously observed [67–71], two A3G mutants, A3G-C97A and A3G-D128K, defective in their Vif-induced degradation, presented a slight or a strong defect in their Vif binding capacity, respectively (Figure 6d, left panel). Concerning the A3G/HDAC6 interaction, we observed that A3G interacted with any of the HDAC6 constructs used in our study (Figure 6e), containing or not the BUZ domain, the BUZ domain alone or a dead-mutant (dm) HDAC6 protein which harbours a double point mutation (H216A/H611A) inactivating deacetylation [44, 55, 65]. This dm-HDAC6 mutant was added in our study because the deacetylase activity of HDAC6 has been shown to be involved in promoting autophagosome-lysosome fusion and removal of toxic aggregates [47–49, 72]. The two A3G mutants, C97A and D128K were able to interact with any of the HDAC6 constructs as occurred with wt-A3G (Figure 6d, right panel). Thus, the binding interface between A3G and HDAC6 could be different from the one from A3G and Vif, as observed with the A3G-D128K mutant. Beside, GST-Vif was able to pull-down all HDAC6 constructs (Figure 6e), thus confirming its direct interaction.

Remarkably, the BUZ domain directly interacts with A3G or Vif (Figure 6e). Data using the HA-HDAC6-∆BUZ construct indicate that regions different from the BUZ domain could also be involved in the HDAC6/A3G or Vif interactions, which could help HDAC6 to compete for the A3G-Vif association thus interfering with the Vif-mediated A3G degradation. Hence, the BUZ domain seems to be responsible for efficient HDAC6/A3G interaction. The H216/H611 residues and subsequent deacetylase activity of HDAC6, it does not seem to be required for the direct HDAC/A3G interaction. Hence, the HDAC6/A3G or HDAC6/Vif appears to be established through different regions of the molecule.

Altogether, these results confirm that the BUZ domain, together with other regions of HDAC6 (excepted the deacetylase domain), are responsible for its interaction with A3G.

Furthermore, we showed that a ternary complex can be formed between HDAC6, A3G and Vif (Figure 6f; and scheme (2) in Figure 6g). Indeed, we showed that Vif could not interact with A3G-D128K (Figure 6d, left panel; and scheme (3) in Figure 6g), contrary to HDAC6 that still interacted with the two A3G mutants (Figure 6d, right panel; and scheme (1) in Figure 6g). By using the GST-A3G-D128K, we observed that this A3G mutant is able to pull-down together HDAC6 and Vif (Figure 6f), thereby indicating that a ternary A3G/HDAC6-Vif complex has been established. Once more, the BUZ domain does not seem to be absolutely required as a ternary complex can still be formed with the HDAC6-∆BUZ construct, even if this protein was less present in the pull-down fractions (Figure 6f, right panel). Taken together, these results account for the ability of HDAC6 to form a ternary complex with A3G and Vif and open-up the possibility that HDAC6 competes for the A3G-Vif interaction, thus protecting A3G from its Vif proteasomal degradation.

### Vif interacts with HDAC6/A3G

**Figure 7:**
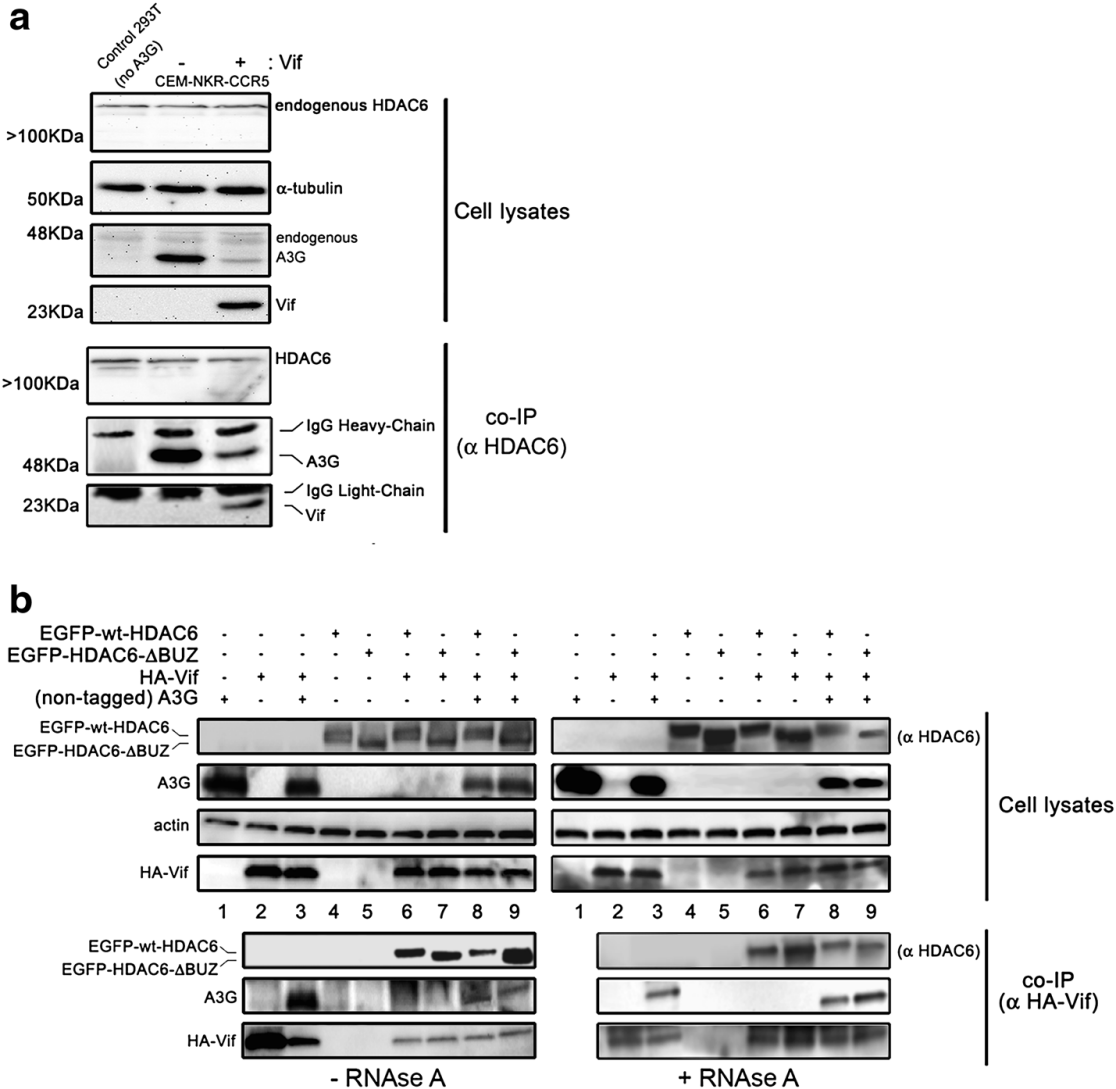
HDAC6 interacts with Vif and A3G in cells. **a** Cell lysates from CEM.NKR.CCR5 cells expressing endogenous A3G and HDAC6 together with overexpressed Vif were subjected to co-IP with anti-HDAC6 Ab, followed by immunoblotting with HDAC6, A3G or Vif Abs. **b** Cell lysates from HEK 293T cells expressing non-tagged A3G alone or co-expressed with HA-Vif, EGFP-wt-HDAC6 or EGFP-HDAC6-ΔBUZ, were subjected to co-IP with anti-HA Ab from cell lysates treated or not with RNAse A (*right and left*, respectively), followed by immunoblotting with HDAC6, A3G or Vif Abs. In **a**, **b**, actin or α-tubulin is the control for total protein, and when detected endogenous HDAC6 and IgG *Heavy*- or *Light-chains* are indicated. Data shown are representative of three independent experiments.

We next analyzed the effect of Vif on the formation of the HDAC6/A3G complex. For this, we co-immunoprecipitated both endogenous HDAC6 and A3G [73, 74] from CEM.NKR.CCR5 cell lysates, in the absence or in the presence of overexpressed Vif protein (Figure 7a). First, these data confirm that A3G and HDAC6 can endogenously interact and form a constitutive complex independently of Vif (Figure 7a). Although overexpression of Vif degraded endogenous A3G, we were able to co-immunoprecipitate non-degraded A3G and Vif proteins with HDAC6. These results indicate that HDAC6 interacts with A3G or with Vif, and suggest that the three molecules may coexist in an HDAC6-mediated complex, as observed above with in vitro recombinant proteins (Figure 6f).

We also seek for a potential HDAC6-Vif binding in HEK 293T cells overexpressing both proteins together with A3G (Figure 7b), and from cell lysates treated or not with RNAse A. We observed that HA-Vif co-immunoprecipitates with wt-HDAC6 or HDAC6-ΔBUZ, whether A3G was present or not, suggesting that HDAC6 and Vif interact in a BUZ-independent manner (Figure 7b, lanes 6 and 7). Moreover, co-immunoprecipitated A3G could correspond to proteins that directly interact with Vif (Vif/A3G) (Figure 7b, lane 3), or indirectly through its interaction with HDAC6 (Vif-A3G/HDAC6 complex) (Figure 7b, lanes 8 and 9). As previously observed (Figure 1d), overexpression of wt-HDAC6 in the presence of Vif induced a slight decrease in A3G expression (Figure 7b, compare lanes 3 and 8 in cell lysates), thus suggesting that HDAC6 forms a complex with A3G that modulates its steady-state expression in the presence of Vif. Moreover, all binary (Vif/A3G and Vif/HDAC6) and ternary (Vif-HDAC6/A3G) interactions observed above were direct as they were also detected from cell lysates treated with RNase A (Figure 7b, right panel), suggesting RNA is not a binding partner. Altogether, these findings suggest that Vif interacts with the HDAC6/A3G complex through its A3G- and/or HDAC6-associated binding sites. The ability of HDAC6 to interact with Vif could account for the above reported HDAC6-mediated degradation of Vif and A3G protection, in a BUZ domain-dependent manner.

### HDAC6 impairs Vif-mediated A3G ubiquitination and proteasome degradation

**Figure 8:**
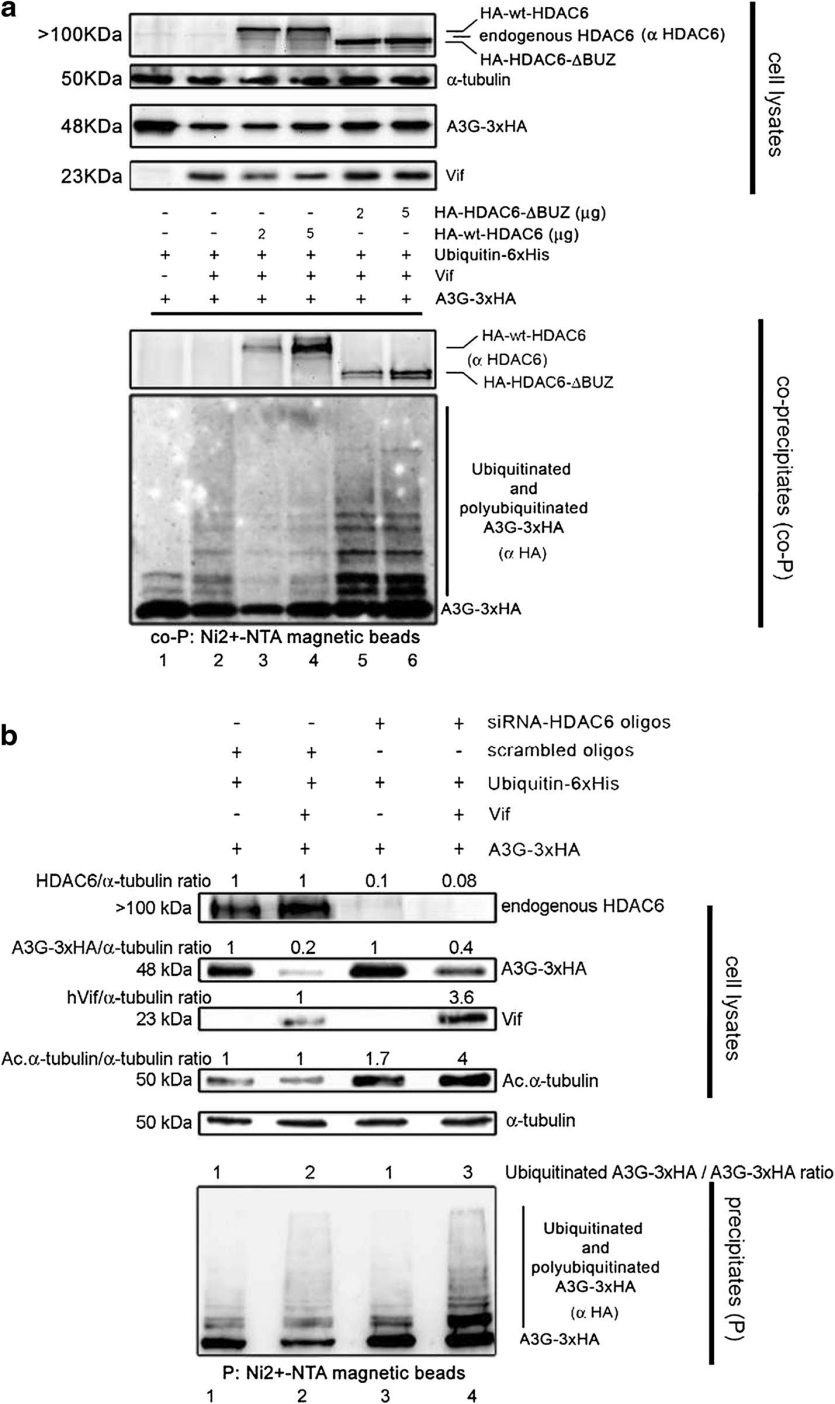
HDAC6 protects A3G from Vif-mediated ubiquitination and degradation in a BUZ domain-dependent manner. **a** Cell lysates from controls expressing A3G-3xHA, or lysates co-expressing A3G-3xHA with Vif, HA-wt-HDAC6 or HA-HDAC6-ΔBUZ were subjected to pull-down with Ni2^+^-NTA magnetic agarose beads to precipitate ubiquitinated proteins coupled to Ubiquitin-6xHis (overexpressed in all experimental conditions). Co-precipitated (co-P) extracts were immunoblotted with specific Abs directed against HA and HDAC6 to monitor co-precipitated ubiquitinated (-6xHis) A3G together with HDAC6 constructs. **b** Cell lysates from control scrambled or siRNA-HDAC6-treated cells, expressing A3G-3xHA with or without Vif, were subjected to pull-down with Ni2^+^-NTA magnetic beads to precipitate ubiquitinated proteins as above. Precipitated (P) extracts were immunoblotted with a specific Ab directed against HA to monitor ubiquitinated (6xHis) A3G. HDAC6/α-tubulin (to quantify silencing efficiency), A3G-3xHA/α-tubulin and (Ubiquitinated A3G-3xHA)/A3G-3xHA (to quantify the increase of A3G ubiquitination) ratios are shown. In **a**, **b**, input expression levels of overexpressed or silenced proteins, as well as endogenous HDAC6 and α-tubulin are shown. Data shown are representative of three independent experiments.

Next, we analyzed the ability of Vif to promote the ubiquitination and degradation of A3G in the presence of wt- or ∆BUZ-HDAC6, after co-transfection in HEK 293T cells together with Ubiquitin-6xHis. This Ubiquitin construct allowed us to pull-down ubiquitinated proteins, and thereby obtain ubiquitinated A3G (Figure 8a). Before pulling-down total ubiquitinated proteins form cell lysates (24 h post-transfection), intact cells were treated with MG132 for 5 h to accumulate Vif-promoted ubiquitinated A3G species. We observed that overexpression of wt-HDAC6 protects A3G from Vif-mediated ubiquitination and degradation in a dose-dependent manner (Figure 8a, lanes 3 and 4). Indeed, we observed a low precipitation level for ubiquitinated A3G in cells overexpressing wt-HDAC6 (Figure 8a, co-P, lanes 3 and 4), as previously described in cells overexpressing HDAC6 where ubiquitinated proteins accumulated in small amounts prior to aggresome deaggregation and clearance [50, 53]. Although HDAC6-ΔBUZ protected A3G against Vif-mediated degradation (Figure 8a, cell lysates lanes 5 and 6, and Figure 1d), it did not impede Vif-mediated ubiquitination of A3G (Figure 8a, co-P, lanes 5 and 6), correlating with the inability of the HDAC6-ΔBUZ mutant to recognize and clear-off ubiquitinated protein aggregates, in contrast to wt-HDAC6 [50].

To further confirm the protective effect exhibited by HDAC6 on Vif-mediated A3G ubiquitination and degradation, we performed similar experiments in which endogenous HDAC6 was silenced by siRNA (Figure 8b). In the absence of Vif, we observed that specific HDAC6 siRNA affected neither A3G expression nor its basal ubiquitination level (Figure 8b, cell lysates and P, compare lanes 1 and 3 without Vif). However, in presence of Vif, specific knockdown of HDAC6 favoured Vif-mediated ubiquitination of A3G (Figure 8b, compare lanes 2 and 4 in P), and did not protect A3G from Vif-mediated degradative activity (Figure 8b, compare lanes 3 and 4 in cell lysates). As previously observed (Figure 2b), specific knockdown of HDAC6 increased acetylation of α-tubulin (Figure 8b, cell lysates). Therefore, HDAC6, through its BUZ domain, partially protects A3G by neutralizing the Vif-mediated A3G ubiquitination and proteasome degradation.

### HDAC6 mediates Vif degradation independently of A3G-Vif interaction, in an autophagy-dependent pathway

**Figure 9:**
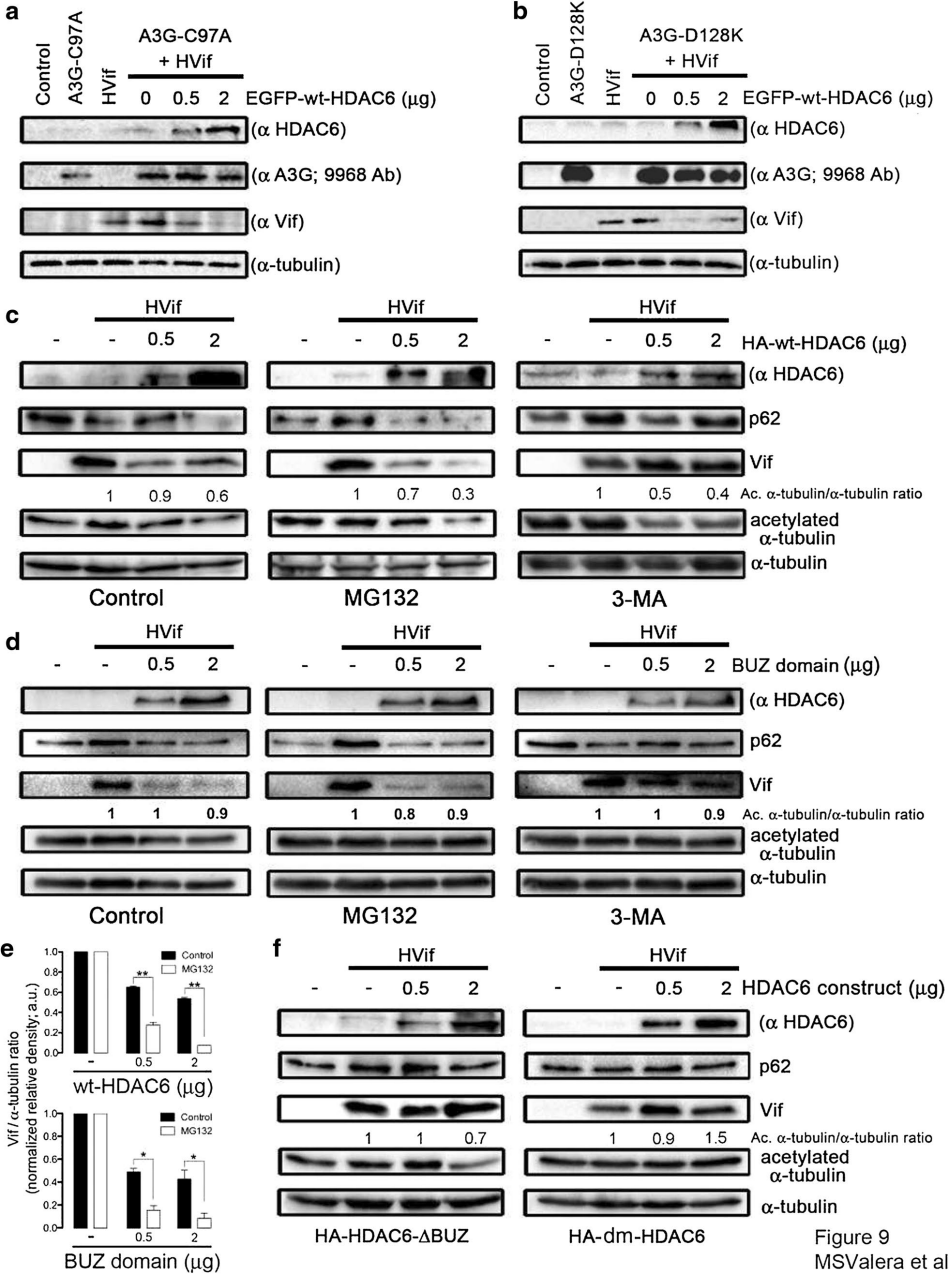
The autophagic degradation of Vif is mediated by the BUZ domain and deacetylase activity of HDAC6. **a**, **b** Western blot of HDAC6-mediated Vif degradation in HEK 293T cells co-expressing the A3G-C97A (**a**) or A3G-D128K (**b**) mutants resistant to Vif. **c**, **d** Western blot of HDAC6- or BUZ-mediated Vif degradation concomitant with p62 analysis in HEK 293T cells co-expressing Vif and HA-wt-HDAC6 or HA-BUZ in the absence of A3G, or in cells treated with MG132 or 3-MA inhibitors. **e** Histograms show quantification of the positive effect exerted by proteasomal inhibition by MG132 on wt-HDAC6- and BUZ-mediated Vif autophagic degradation. Data are from three independent western-blot of wt-HDAC6 and BUZ domain constructs, as presented in (**c**–**d**), comparing Vif/α-tubulin ratios in Control and MG132 experimental conditions. Data are mean ± S.E.M. of three independent experiments carried out in triplicate: ** and * are p < 0.01 and p < 0.05, respectively, Student’s *t* test. **f** Western blot of HA-HDAC6-ΔBUZ- and HA-dm-HDAC6-effect on Vif and p62 stability in HEK 293T cells in the absence of A3G. From **a** to **f**, when indicated, control untreated cells, cells only expressing Vif or each A3G construct or endogenous HDAC6 are shown. When indicated, HDAC6 constructs-deacetylase activity against acetylated α-tubulin is shown. α-tubulin is the control for total protein. Data are representative of three independent experiments.

Our data indicate that HDAC6 promotes Vif degradation, and protects A3G from its Vif-triggered proteasome degradation. We next analyzed the ability of HDAC6 to promote Vif degradation in the presence of two A3G mutants, such as A3G-C97A and A3G-D128K, which were reported to be resistant to Vif-mediated proteasome degradation [67–71]. We observed that HDAC6 promoted Vif degradation in the presence of A3G-C97A or A3G-D128K mutants, which in turn were not degraded by Vif in cells that did not overexpress wt-HDAC6 (Figure 9a, b), suggesting that HDAC6 promotes Vif degradation independently of the ability of Vif to interact and/or degrade A3G.

To this end, we studied the degradative pathway triggered by HDAC6, which clears-off Vif in the absence of A3G. We observed that HDAC6-mediated Vif degradation is blocked by 3-methyladenine (3-MA), an inhibitor for autophagic sequestration of cytoplasmic proteins [75–77] (Figure 9c, right panel). On the contrary, overexpression of HDAC6 in untreated or MG132-treated cells triggered Vif degradation, and promoted degradation of p62 or sequestosome 1 (SQSTM1) (Figure 9c). This p62/SQSTM1 protein (hereafter designated p62) connects ubiquitinated protein aggregates to autophagosomes, and also facilitates their clearance [78, 79]. Interestingly, an HDAC6 construct only containing the C-terminal BUZ domain was also able to induce Vif degradation and promote p62 decay (Figure 9d, left panel). This degradation was inhibited by 3-MA but not by MG132 (Figure 9d). Although the MG132 inhibitor did not impede HDAC6-triggered degradation of Vif, it favours the autophagic removal of Vif by either wt-HDAC6 or the C-terminal BUZ domain construct (Figure 9c, d; quantified in Figure 9e). MG132 treatment of cells could favor the HDAC6-mediated autophagic degradation of Vif, as we observed. Remarkably, we did not observed p62 decay and Vif degradation in cells overexpressing HDAC6-ΔBUZ (Figure 9f, left panel). Although wt-HDAC6 and HDAC6-ΔBUZ constructs were enzymatically functional as shown by the onset of α-tubulin deacetylation (Figure 9c, f), only the HDAC6-ΔBUZ construct was unable to degrade Vif (Figure 9f).

Next, we asked whether the deacetylase activity of HDAC6 was required to induce the reduction of Vif, as deacetylation was reported to be involved in promoting autophagosome-lysosome fusion and removal of toxic aggregates [49, 50]. Indeed, we observed that the deacetylase mutant [(dm)-HDAC6] did not induce Vif and p62 degradation, in accordance with a decoupling of Vif from the aggresome-autophagy degradation pathway (Figure 9f, right panel). Taken together, our results suggest that Vif is removed in an HDAC6-dependent autophagic pathway requiring its C-terminal BUZ domain and deacetylase activity.

### HDAC6-mediated proviral Vif degradation inhibits HIV-1 infectiveness

**Figure 10:**
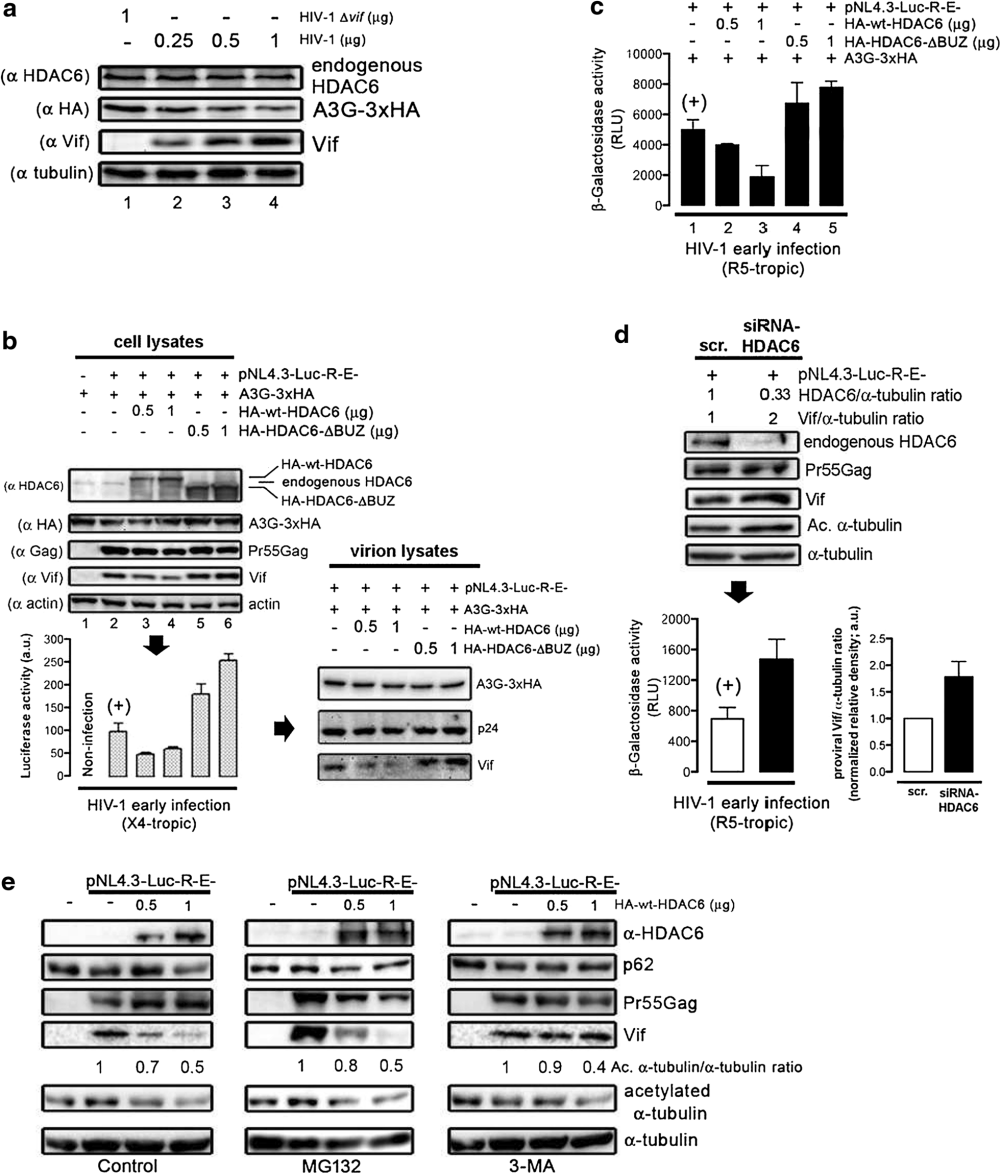
HDAC6-mediated proviral Vif degradation stabilizes A3G and impairs HIV-1 infectiveness. **a** Western blot of A3G degradation by proviral Vif in HEK 293T cells co-transduced with A3G-3xHA and HIV-1_NL4.3_ (dose–response) or HIV-1 *Δvif* provirus. **b** Effect of HDAC6 on HIV-1 infectiveness assayed in HeLa P5 cells incubated with equivalent luciferase-based X4-tropic pNL4-3.Luc.R-E- viral inputs produced in control, or in HEK 293T cells overexpressing A3G-3xHA, or overexpressing HA-wt-HDAC6 or HA-HDAC6-ΔBUZ together with A3G-3xHA. The control of infection (+) is represented by virus produced in HEK 293T, only expressing A3G-3xHA. A3G-3xHA and Vif proteins incorporated into nascent virions (p24) are shown. **c** Effect of HDAC6 on HIV-1 infectiveness, assayed in HeLa P5 cells incubated with equivalent R5-tropic pNL4-3.Luc.R-E- viral inputs [same experimental conditions as in (**a**)], and measured by β-galactosidase activity. **d** Effect of specific HDAC6 siRNA on HIV-1 infectiveness assayed in HeLa P5 cells incubated with equivalent R5-tropic viral inputs produced in siRNA-HDAC6-treated HEK 293T cells, and compared to control (+), scrambled-treated HEK 293T cells. HDAC6/α-tubulin and proviral-Vif/α-tubulin expression ratios are shown for this representative experiment. Vif/α-tubulin ratios from three independent experiments are shown in the right histogram. **e** Western blot of HDAC6-mediated proviral Vif degradation concomitant with p62, in control HEK 293T cells transduced or not with a pNL4.3-Luc-R-E- provirus, together with HA-wt-HDAC6, in the absence of A3G. 3-MA or MG132 treatment is shown under the same conditions. Tubulin-deacetylase activity of HDAC6, and proviral Pr55^Gag^ and Vif proteins are shown. All western blots are from a representative experiment of three, and show input expression for endogenous HDAC6 (in control, scrambled- or siRNA-HDAC6-treated cells), overexpressed HA-wt-HDAC6 or HA-HDAC6-ΔBUZ, or A3G-3xHA and proviral Pr55^Gag^, p24 and Vif proteins. Infection assay were done in triplicate and results are representative of four independent experiments (mean ± S.E.M.; n = 12). When indicated, α-tubulin or actin is the control for total protein.

Next, we analyzed the ability of HDAC6 to degrade HIV-1 proviral Vif and its effect on HIV-1 infectiveness. First, in HEK 293T cells overexpressing A3G, we observed that Vif expressed from pNL4.3 HIV-1 provirus degraded A3G in a dose-dependent manner (Figure 10a), using as a control a provirus lacking the *vif* gene. Then, we quantified the effects of HDAC6-mediated proviral Vif degradation on early HIV-1 entry and infection by using a pNL4.3.LucR-E- provirus (bearing the *lacZ* gene) (Figure 10b) [80–83]. The overexpression of pNL4.3.LucR-E- in packaging HEK 293T cells, in conjunction with an X4-tropic envelope (Env) induced a decrease of A3G (Figure 10b, compare lane 2 with control lane 1). This effect was Vif-dependent since overexpression of wt-HDAC6 promoted proviral Vif decrease and subsequent A3G protection in a dose-dependent manner (Figure 10b, lanes 3 and 4). As observed above (Figure 1d), HDAC6-ΔBUZ protected A3G from proviral Vif-mediated degradation, and was unable to clear off Vif (Figure 10b, lanes 5 and 6).

Free-cell X4-tropic virions produced under these conditions were collected to infect permissive HeLa P5 cells with viral synchronous doses (Figure 10b, histograms). We observed that HIV-1 early infection was impaired when wt-HDAC6 was overexpressed in packaging HEK 293T cells, thus corresponding to conditions where Vif was degraded and A3G subsequently protected (Figure 10b, histograms, compare control bar in lane 2 with lanes 3 and 4). On the contrary, HIV-1 early infection was enhanced with viruses produced in HEK 293T cells overexpressing HDAC6-ΔBUZ, where Vif level was increased and A3G apparently protected from Vif degradation (Figure 10b, histograms, bars in lanes 5 and 6), suggesting that HDAC6-ΔBUZ competes with the anti-HIV-1 activity of the endogenous HDAC6, thus controlling Vif expression level and infectiveness (Figure 10b, compare Vif and infection between lane 2 and lanes 5–6). Indeed, we observed a strong correlation between the amount of Vif in cells producing viruses and in nascent virions under these experimental conditions (Figure 10b, virion lysates). Surprisingly, HIV-1 infectiveness was not correlated to A3G level, apparently stabilized by overexpressed wt-HDAC6 or HDAC6-ΔBUZ (Figure 10b). Therefore, it seems that HIV-1 infectiveness correlates with Vif stability.

The anti-HIV-1 effect of HDAC6 was similarly confirmed with R5-tropic virions produced in HEK 293T cells with R5-tropic HIV-1 Env, under the same experimental conditions (Figure 10c). Indeed, virions produced with overexpressed wt-HDAC6 were less infectious than control virions or virions produced in the presence of HDAC6-∆BUZ (Figure 10c). Accordingly, HIV-1 infectiveness was enhanced in virions produced in HEK 293T cells where endogenous HDAC6 was silenced, in correlation with an increase amount of Vif (Figure 10d). Thus, there is a clear-cut and inverse correlation between the HDAC6 level and its degradative activity against Vif, and HIV-1 infectiveness.

To ascertain the HDAC6-triggered degradative pathway for Vif, we analyzed the expression of Vif in HEK 293T cells treated with proteasome and autophagosome/autophagy inhibitors. We observed that the HDAC6-mediated Vif degradation was blocked by 3-MA but not by MG132 (Figure 10e), without affecting its deacetylase activity (Figure 10e, Ac. α-tubulin). HDAC6 overexpression appeared to promote autophagy, as evidenced by a low p62 steady-state level and restored by 3-MA treatment (Figure 10e, see also Figure 9c), suggesting that HDAC6 clears-off Vif by inducing its autophagic degradation.

## Discussion

In this study, we report for the first time that HDAC6 represents a new cellular factor involved in HIV-1 restriction. We showed that HDAC6 directly interacts with A3G and forms a constitutive HDAC6/A3G complex co-distributing in the cytoplasm and along MTs, as expected from the ability of HDAC6 to bind MTs, either directly or through its association with MT-motor machinery [45]. Then, the binding of HDAC6 to A3G or to Vif counteracts Vif activity, which in turn is unable to degrade A3G. Although the C-terminal BUZ domain of HDAC6 appears to be dispensable for HDAC6/A3G interaction, this C-terminal region is required to promote its clearance through autophagy. Moreover, HDAC6 targets Vif, without affecting its CBF-β E3 ligase cofactor, in a BUZ-dependent manner. HDAC6 could exert an anti-HIV-1 activity by stabilizing A3G by means of affecting Vif and the subsequent assembly of the Vif-Cul5 E3-ubiquitin-ligase complex that degrades A3G [36, 38]. In fact, interfering with the Vif-CBF-β interaction destabilizes the formation and degradative activity of the Vif-Cul5 E3-ubiquitin-ligase complex, as observed for some EloB mutants [38]. Similarly, the apoptosis signal-regulating kinase 1 (ASK1) has been shown to target Vif and block its interaction with EloB/C, thus affecting the E3 ligase complex formation and function [84]. HDAC6 appears therefore as a new natural factor that protects A3G by interacting with and degrading Vif.

The HDAC6/A3G interaction also occurred in presence of Vif, and resulted in a ternary A3G/HDAC6-Vif complex that could also be found in a BUZ-independent manner. Similarly, in the absence of A3G, Vif directly interacts with HDAC6 in a BUZ-independent manner. Therefore, it is conceivable that HDAC6/A3G, HDAC6-Vif and the ternary A3G/HDAC6-Vif complexes may be established by different binding sites, as observed by the ability of various HDAC6 mutants and the BUZ domain to interact with A3G or Vif, being the BUZ domain responsible for the HDAC6-mediated degradation of Vif. Remarkably, the BUZ domain directly interacts with A3G or Vif (Figure 6e), thus confirming that HDAC6 could compete for the A3G-Vif interaction and target Vif for HDAC6-mediated aggresome-autophagic degradation, rescuing A3G in a BUZ dependent manner.

The HDAC6/A3G, HDAC6-Vif or HDAC6/A3G-Vif associations are established by direct protein–protein interactions and seems not to be mediated by the interplay of CBF-β, of any nucleic acids that could be targeted by Vif and/or A3G just harbouring HDAC6 into the different complexes. Hence, direct interaction and complexes described here were confirmed by a GST-based in vitro protein–protein interaction system, by using protein recombinant-mediated pull-down experiments, and from cell lysates treated with RNAse A.

Additionally, we showed that HDAC6 protects A3G from Vif-mediated ubiquitination and degradation in a dose-dependent manner. Hence, we observed low precipitation levels for ubiquitinated A3G in cells overexpressing HDAC6, as previously reported for ubiquitinated aggregates [50, 53]. Although HDAC6-ΔBUZ interacts and co-distributes with A3G, and appears to protect A3G against Vif-mediated degradation, it does not abrogate Vif-mediated ubiquitination of A3G. This could correlate with the fact that HDAC6-ΔBUZ is unable to bind free or protein-associated ubiquitin, or to clear ubiquitinated protein aggregates compared to wt-HDAC6 [50]. Similar defects have been described with C-terminal point mutants disrupting the ability of HDAC6 to bind ubiquitin [51, 53, 58]. We observed, in presence of Vif, that knockdown of HDAC6 favoured Vif-mediated ubiquitination and degradation of A3G, suggesting that endogenous HDAC6 exerts anti-Vif activity. Thus, HDAC6 could protect A3G from Vif-mediated degradation by two cooperative actions: (1) by directly triggering Vif degradation in a BUZ-dependent manner, a process that it is also favoured by the HDAC6-deacetylase activity; and (2) by forming an HDAC6/A3G complex thus decoupling A3G from the Vif-associated proteasomal degradation pathway (see Additional file 1: Summary illustration). This is exemplified by the HDAC6-ΔBUZ construct that inhibited Vif-mediated proteasomal degradation of A3G but failed to impair A3G ubiquitination by Vif. Thus, HDAC6 appears to stabilize non-ubiquitinated A3G molecules by forming an HDAC6/A3G complex. In this way, it controls the level of A3G expression, and clear ubiquitinated A3G molecules in a dose-dependent manner. The ability of HDAC6 to control A3G steady-state expression through an HDAC6/A3G complex could be important for A3G immune functions, which are known to occur when the editing enzyme is expressed at high levels, and/or in those infectious environments where Vif is depleted from virions [3, 12, 21, 26, 85–87].

The clearance of Vif by HDAC6 occurs whether Vif is expressed from an expression vector or from a proviral construct, and whether A3G is expressed or not. Moreover, HDAC6-mediated proviral Vif degradation is insensitive to MG132, but is blocked by 3-MA, an inhibitor for autophagic sequestration of cytoplasmic proteins that act on class III PI3 kinase complex [75–77]. HDAC6 also promotes degradation of p62, a molecule connecting ubiquitinated protein aggregates to autophagosomes to facilitate their clearance [78, 79]. The p62 protein becomes incorporated into completed autophagosomes and is degraded in autolysosomes, where inhibition of autophagy correlates with increased p62 levels, suggesting that steady-state levels of this protein reflect an autophagic status [77]. In contrast, HDAC6-ΔBUZ does not trigger p62 clearance and seems to stabilize and increase Vif expression, confirming the importance of HDAC6 in the clearance of ubiquitinated protein-aggregate in a BUZ domain-dependent manner [47, 49–51]. These processes could be perturbed by 3-MA that alter the formation of the protein-sequestering autophagosome compartment [75–77, 88], thus explaining its inhibitory effect on HDAC6-mediated Vif degradation (Figure 9c). In cells where proteasomal clearance is blocked, protein-free polyubiquitin chains are formed that could be recognized by HDAC6. This event activates HDAC6-triggered actinomyosin- and autophagy-dependent aggresome processing of proteins [53], and could account for the MG132-mediated increase in the ability of wt-HDAC6 and the BUZ domain to degrade Vif by autophagia.

It has been reported that the deacetylase activity of HDAC6 is important in promoting autophasome-lysosome fusion and removal of toxic aggregates [47–49], thus deacetylase mutants of HDAC6 decouple target substrates from the autophagy-mediated degradation pathway. Here, we showed that the inactivation of the deacetylase domain of HDAC6 (mutant H216A/H611A) not only affects the deacetylation of α-tubulin [44, 55, 65], but also perturb the degradation of Vif by autophagy (Figure 9d), indicating that the deacetylase activity, in conjunction with the BUZ domain of HDAC6, helps the enzyme to form aggresomes and clears-off Vif by autophagy. This effect seems to be independent on the HDAC6/Vif interaction as HDAC6 constructs lacking the BUZ or the deacetylase motifs still interact with Vif (Figures 6b, 7b).

Vif uses both degradation-dependent and -independent mechanisms to counteract A3G antiviral activity. Indeed, Vif has been shown to block the encapsidation of an A3G mutant (A3G-C97A) without any neutralisation/degradation of this mutant by Vif in cell [67]. Here, we showed that HDAC6 was still able to promote the degradation of Vif in presence of A3G-C97A and A3G-D128K mutants, both resistant to Vif-mediated proteasome degradation. Of note and as previously observed [67–71], (1) both mutants are resistant to Vif-mediated proteasome degradation (Figure 9a), and (2) the C97A substitution, which abrogates A3G multimerization, does not affect its interaction with Vif (Figure 6d), while the D128K substitution can no longer interact with Vif (Figure 6d–f). Thus, HDAC6 could protect wt- or A3G mutants and degrade Vif independently on the ability of Vif to degrade and interact with A3G.

In fact, we observed a clear-cut correlation between the HDAC6 ability to degrade HIV-1 Vif and the restoration of A3G level in cells producing virions and HIV-1 infectiveness, regardless viral tropism. Indeed, HIV-1 infection was impaired when viral particles were produced in HEK 293T cells overexpressing wt-HDAC6, where Vif is degraded and A3G subsequently protected. On the contrary, infectivity of viruses produced in HEK 293T cells overexpressing HDAC6-ΔBUZ was enhanced. This effect was not completely surprising as the BUZ domain of HDAC6 is required for the autophagy-mediated degradation of Vif. Moreover, the fact that ubiquitinated A3G are increased when the BUZ domain is absent (Figure 8a) suggests these A3G species are not functional in their restrictive activity against HIV-1 (Figure 9b). Therefore, it appears that HIV-1 infectiveness correlates with the stability of Vif, which is inversely dependent on HDAC6 expression level, rather than on the level of A3G expression. In the present work, we report that the amount of Vif incorporated into nascent virions correlates with their infectious activity, being these events directly dependent on the level of HDAC6 expression and on its ability to promote Vif autophagic degradation (Figure 10b). Thus, HIV-1 infectious activity is directly related to the level of Vif incorporated into nascent virions, rather than the presence of A3G (Figure 10b), as recently reported [89]. Of note, Vif level increased with HDAC6-ΔBUZ either in cells producing virions or into nascent virions, thereby generating more infectious virions than those derived from control cells. The same holds true when endogenous HDAC6 was knocked-down (Figure 10d). On the contrary, HDAC6 promotes Vif degradation and impairs its incorporation into nascent virions, thereby diminishing viral infectivity (Figure 10b, c). Altogether these data indicate that HDAC6 is controlling the infectiveness of nascent viral particles by compromising the amount of Vif incorporated into the virions, a process that is independent of the presence of A3G. Consequently, the anti-viral function of HDAC6 could be overcome by an increased expression level of Vif during viral replication.

Considering that (i) HIV-1 stabilizes acetylated microtubules during the initial HIV-1 Env/CD4 contacts to favour pore fusion formation and infection [44, 46, 90], and (ii) HDAC6 plays an anti-HIV-1 replicative activity regulating this process [44–46], in conjunction with the impairment of both Tat proviral function (by HDAC6-mediated Tat deacetylation) [91] and the proposed HDAC6/A3G restriction complex, the regulation of HDAC6 in immune cells could represent a new way to overcome HIV-1 Vif proviral functions and control HIV-1 infectiveness. It is plausible that the amount of endogenous HDAC6 conditions the permissibility of cells against HIV-1 infection, first by exerting its above-reported antiviral actions, and second, by efficiently forming the physiological HDAC6/A3G complex, essential for promoting A3G and HDAC6 anti-viral functions.

## Conclusions

These findings led us to propose that HDAC6 and HDAC6/A3G may represent a natural antiviral factor and complex, respectively. They would be capable to protect the antiviral activities of A3G and promote the autophagic degradation of Vif, thus impairing its incorporation into nascent virions and HIV-1 infectiousness (see Additional file 1: Summary illustration). We propose HDAC6 as a new natural factor that restricts HIV-1 infection.

## Methods

### Antibodies and reagents

Rabbit anti-HDAC6 (H-300; c-11420), anti-ubiquitin (FL-76; sc-9133), anti-EGFP (FL; sc-8334) and anti-HA (sc-805) polyclonal antibodies (polyAbs), goat APOBEC3G (Q17; sc-27521) polyAb, and mouse anti-HIV-1 Vif (319; sc-69731), anti-HA-probe (F-7; sc-7392), anti-c-myc (9E10; sc-40), and anti-Hsp90α/β (F-8; sc-13119), anti-CBF-β (sc-56751) and anti-p62/SQSTM1 (D-3; sc28359) monoclonal Abs (mAbs), or rabbit anti-acetylated lysine (AB3879) polyAb were obtained from Santa Cruz Biotechnology (Santa Cruz, CA, USA), or Millipore (Millipore Corporation, Billerica, MA, USA), respectively. Rabbit anti-(HIV1 p55 + p24 + p17) (ab63917) polyAb was purchased from Abcam (Cambridge Science Park, Cambridge, UK). Mouse anti-α-tubulin (clone B-5-1-2), anti-acetylated tubulin (clone 6-11B-1), anti-β-actin (clone AC-74; A2228) and anti-Flag M2 (clone M2; F1804) mAbs, and anti-Flag (F7425) and anti-HA (H6908) rabbit polyAbs were obtained from Sigma-Aldrich (Sigma-Aldrich, St. Louis, USA). Secondary horseradish peroxidase (HRP)-conjugated antibodies, specific for any antibody species assayed, were purchased from Dako (Glostrup, Denmark), while Alexa Fluor 568-labeled donkey anti-goat, Alexa Fluor 488-labelled donkey-anti-rabbit, Alexa Fluor 633-labelled goat-anti-mouse, Alexa Fluor 568-labeled goat-anti-rabbit, Alexa Fluor 633-labelled rabbit-anti-mouse, Alexa Fluor 568-labeled donkey-anti-rabbit, Alexa Fluor 488-labeled rabbit-anti-goat were purchased from Molecular Probes (Eugene, OR, USA). Neutralizing mAb RPA-T4 (eBioscience, San Diego, CA, USA) was directed against CD4 and used to inhibit HIV-1 entry and infection. Endotoxin-free, recombinant soluble (rs) X4-tropic HIV-1 Env-gp120-IIIB protein (rs-gp120_IIIB_) was produced in *Escherichia coli* by Innogenetics (Ghent, Belgium) and used as previously described [80, 81, 92]. MG132 (Z-Leu-Leu-Leu-al), ALLN and 3-methyladenine (3-MA) inhibitors were acquired from Sigma-Aldrich. Complete 25X and Phostop 10X inhibitors and the RNAse A enzyme were obtained from Roche Diagnostics (GmbH, Mannheim, Germany). TNT^®^ Quick Coupled Transcription/Translation System (Promega BioSciences, LLC; CA, USA) was used for in vitro production of recombinant proteins. Monoclonal Anti-HA-Agarose antibody for recombinant protein purification was purchased from Sigma-Aldrich. Glutathione-Sepharose and pGEX-4T1 were from Amersham Biosciences (NJ, USA). Anti-HA monoclonal antibody (clone 16B12) was purchased by Covance Antibodies (Covance, MA, USA), whereas mouse monoclonal anti-GST antibody (sc-138) was from Santa Cruz Biotechnology. For Western blotting, secondary horseradish peroxidase-conjugated anti-IgGs were from Pierce Antibodies (Thermo Fisher Scientific Inc., NYSE: TMO).

### Cells

HEK 293T cells was grown at 37°C in a humidified atmosphere with 5% CO_2_ in DMEM (Lonza, Verviers, Belgium) supplemented with 10% foetal calf serum (Lonza), 1% l-glutamine and 1% penicillin–streptomycin (Lonza). Cells were harvested, passaged every 3 days, and cultured to 50–70% confluence in fresh supplemented DMEM 24 h before cell transfection with viral or human DNA constructs. The HeLa-P5 cells stably transfected with human CD4 and CCR5 cDNAs and with an HIV-long terminal repeat-driven β-galactosidase reporter gene was provided by Dr. M. Alizon (Hôpital Cochin, Paris, France) and cultured as previously described [44]. The human CEM.NKR-CCR5 permissive cell line (catalog number 4376, NIH AIDS Research and Reference Reagent Program) was grown at 37°C in a humidified atmosphere with 5% CO_2_ in RPMI 1640 medium (Lonza) supplemented with 10% fetal calf serum (FCS) (Lonza), 1% l-glutamine, and 1% penicillin–streptomycin antibiotics.

### DNA plasmids and Viral DNA constructs

Drs. X.-J. Yang and N. R. Bertos (Molecular Oncology Group, Department of Medicine, McGill University Health Centre, Montreal, Quebec, Canada) kindly provided all HDAC6 constructs used in the present study [56]. When indicated, these plasmids were cloned into pEGFP-C1 (Clontech, Palo Alto, CA, USA) or N-terminal tagged with the terminal influenza hemagglutinin (HA) epitope. The pcDNA3.1 (Life Technologies) or pEGFP-C1 (Clontech) vectors were used as a control of cDNA transfection or to express free EGFP, respectively. APOBEC3G (A3G) was kindly provided by Dr. B. R. Cullen (Duke University, USA). A3G open-reading frame (KpnI/EcoRI PCR fragment) was cloned first in frame into a pcDNA 3.1-based vector, modified to encode carboxy-terminal triple HA (3xHA), myc or Flag epitope tags. A3G-C97A and A3G-D128K expression vectors were obtained from wt-A3G after site-directed mutagenesis according to the manufacturer (QuickChange mutagenesis, Agilent Technology). They were subsequently excised using *Hind*III and *Xho*I restriction enzymes and cloned into the pK expression plasmid. A plasmid coding for Ubiquitin-6XHis was kindly provided by Dr. M. S. Rodriguez (Ubiquitin-like proteins & Cancer Group. Proteomics Unit. CIC bioGUNE. Parque Tecnológico de Vizcaya, Spain). The pNL4-3-HIV-1 and pNL4-3.Luc.R-E- provirus (catalog number 6070013), the X4-tropic HXB2-*envelope* (*env*, catalogue number 5040154) and R5-tropic pCAGGS-SF162-gp160-*env* (catalogue number 3041817) glycoprotein vectors were obtained via the NIH AIDS Research and Reference Reagent Programme. The HIV-1 pNL4-3 wild-type and *Δvif* proviruses were kindly provided by Dr. J. Blanco (Fundació irsiCaixa-HIVACAT, Institut de Recerca en Ciències de la Salut Germans Trias i Pujol, Hospital Germans Trias i Pujol, Universitat Autònoma de Barcelona, Barcelona, Catalonia, Spain) and by Dr. O. Schwartz (Pasteur Institute, Paris, France), respectively. The pcDNA-hVif plasmid was a gift from Dr. K. Strebel (National Institute of Allergy and Infectious Diseases, Bethesda, USA). Full-length A3G and HIV-1-Vif cDNAs were obtained by PCR using the respective oligonucleotides: A3G 5′-TATATTGAATTCATGAAGCCTCACTTCAGAAACAC-3′ and 5′-TATATTCTCGAGTCAGTTTTCCTGATTCTGGAGAATG-3′; Vif 5′-TATATTGGATCCATGGAGAACCGGTGGCAGGTG-3′ and 5′-TATATTCTCGAGCTAGTGTCCATTCATTGTATGGC-3′. The A3G, A3G-C97A, A3G-D128K or Vif PCR products were cloned into pGEX-4T1 (GE), and then digested by EcoRI/XhoI and BamHI/XhoI, respectively.

### Messenger RNA silencing

Three double-stranded short interference RNA (siRNA) oligonucleotides (oligos) were generated (Eurogentec, Hampshire, United Kingdom) against the mRNA sequence of HDAC6 and spanning nucleotides 193–213, 217–237, and 284–304, as described previously [44]. One day before nucleofection, HEK 293T cells were grown at 37°C in 10 cm^2^ plates to reach 60–80% confluence. Cells were then transfected with a mix of these siRNAs against HDAC6 (in this work referred to as siRNA-HDAC6), at a final total concentration of 1–1.6 μM, or by scrambled-control oligos (1–1.6 μM) using an Amaxa nucleofector kit, an Amaxa cuvette-nucleofector device, and a Q-001 program (Amaxa, Lonza). Nucleofected cells from each experimental condition were recovered in 1 mL of DMEM medium and divided in two 10-cm^2^ plates per condition in a total volume medium of 10 mL, and kept overnight at 37°C. To analyze HDAC6 silencing, whole cell lysates (40 μg) of transfected cells were analyzed by western blot using a specific anti-HDAC6 polyAb.

### Immunofluorescence

HEK 293T cells (2 × 10^6^) were transfected with A3G-3xHA (1 μg), or EGFP-wt-HDAC6 (1 μg) and A3G-3xHA (1 μg), or HA-wt-HDAC6 (1 μg) or HA-HDAC6-ΔBUZ (1 μg) and A3G-3xHA or A3G-Flag (1 μg) in order to analyze the co-distribution of endogenous HDAC6 with overexpressed A3G or the co-distribution of each overexpressed HDAC6 construct with A3G, respectively. To transfect cells, we used 25 kDa (PEI25k) linear polyethylenimine (Polyscience Inc., Warrington, PA, USA), as previously reported [82, 83]. Briefly, HDAC6 and/or A3G plasmids were first dissolved in 1/10th of the final tissue culture volume of DMEM free of serum and antibiotics. PEI25k was prepared at 1 mg/mL solution in water, and adjusted to neutral pH. After adding PEI25k to the plasmids [*plasmids:PEI25k* ratio of *1:3* (w/w)], the solution was mixed immediately, incubated for 30 min at room temperature, and added to HEK 293T cells in culture. After 4 h, medium was changed to fresh DMEM supplemented with 10% fetal calf serum and antibiotics, and cells were cultivated to 60–70% confluence 24 h after transfection. They were then plated on sterile glass coverslips [Ø, 12 mm; previously coated with poly-d-Lysine (0.01% in sterile H_2_O)] and kept in culture for 24 h at 37°C. For immunolabelling, cells on sterile glass coverslips were washed three times with PBS and fixed for 5 min in 3% paraformaldehyde in PBS. Cells were again washed three times with PBS and permeabilized with 0.1% Triton X-100 in PBS. After washing with PBS, cells were immunostained; in Figure 4a, with Alexa Fluor 568-labeled donkey anti-goat against A3G (previously incubated with a specific goat polyAb) to detect overexpressed A3G-3xHA; then with Alexa Fluor 488-labelled donkey-anti-rabbit against HDAC6 (previously incubated with a specific rabbit polyAb) to detect endogenous HDAC6. Endogenous α-tubulin was finally detected by an Alexa Fluor 633-labelled goat-anti-mouse against α-tubulin (previously incubated with a specific mAb). In Figure 4b, cells were immunostained with Alexa Fluor 568-labeled goat-anti-rabbit against HA (previously incubated with a specific rabbit polyAb) to detect overexpressed A3G-3xHA; then with Alexa Fluor 633-labelled rabbit-anti-mouse against α-tubulin (previously incubated with a specific mAb). Overexpressed EGFP-wt-HDAC6 was monitored by EGFP-associated green fluorescence. In Figure 4c, cells were immunostained with Alexa 568-labeled donkey-anti-rabbit against Flag (previously incubated with a specific rabbit polyAb) to detect overexpressed A3G-Flag; then with Alexa Fluor 488-labeled rabbit-anti-goat against HA (previously incubated with a specific goat polyAb) to detect HA-wt-HDAC6 or HA-HDAC6-ΔBUZ. Finally, cells were immunostained with Alexa Fluor 633-labelled rabbit-anti-mouse against α-tubulin (previously incubated with a specific mAb). Coverslips were mounted in Mowiol-antifade (Dako) and imaged in xy midsections with a FluoView FV1000 confocal microscope using a 1.35 NA objective (60×; Olympus, Center Valley, PA, USA) for high-resolution imaging of fixed cells. Final images were analyzed with MetaMorph software (Universal Imaging, Downington, PA, USA), as previously reported [83, 92].

### Western blotting

Protein expression was determined in western blots of cell lysates. Briefly, non-treated HEK 293T cells (2 × 10^6^) or those post-24 h nucleofection, were washed twice in phosphate-buffered saline (PBS) 1X and lysed in cold lysis buffer [containing 1% Triton-X-100, 50 mM Tris–HCl pH 7,5, 150 mM NaCl, 0,5% sodium deoxycholate, and protease/phosphatase inhibitors (Roche) at 1X], for 30 min and sonicated twice for 30 s at +4°C. To analyze by western blot the effects of different inhibitors on HDAC6-triggered Vif (recombinant hVif or proviral Vif) or endogenous CFB-β stability, we similarly assayed HEK 293T cells 24 h post-transfection, then treated for 5 h at 37°C with either MG132 [20 μM; DMSO (dimethyl sulfoxide) was the control vehicle] to inhibit the proteasome, or 3-MA (5 mM; PBS for control samples) to inhibit autophagosome formation and subsequent autophagic degradation, monitored by detecting p62/SQSTM1 protein. Cells were then washed twice in PBS 1X, lysed in cold lysis buffer for 30 min, and sonicated twice for 30 s at +4°C. Equivalent amounts of protein (40 μg), measured using the bicinchoninic acid method, were separated by SDS-PAGE on 12% gradient gels and electroblotted onto 0.45 μm polyvinylidene difluoride membranes (PVDF; Millipore) using Trans-blot Turbo (Bio-Rad, Hercules, CA, USA). Membranes were probed with specific antibodies and the proteins recognised were detected by luminescence using Clarity Western ECL Substrate (Bio-Rad). They were then analyzed using a ChemiDoc MP device and Image LabTM Software, version 4.0.1 (Bio-Rad).

### Immunoprecipitation assays

HEK 293T cells (2 × 10^6^) were co-transfected with different plasmids (1 μg) using PEI25k as indicated above, and using non-tagged A3G, A3G-3xHA, A3G-Flag, A3G-myc, HA-Vif (0.25 μg), and/or each HA- or EGFP-tagged HDAC6 construct indicated, and pcDNA3.1 or pEGFP-C1 (0.5 μg) plasmid as a control condition. For ubiquitination experiments, when indicated, MG132 (20 μM) inhibitor and its DMSO-vehicle control were used. 24 h post-transfection, cells (1 × 10^6^/experimental condition) were washed twice in PBS 1X and lysed in RIPA 1X (PBS 1X, 1% NP40, 0.5% sodium deoxycholate, 0.05% SDS) supplemented with protease inhibitors (complete EDTA Free cocktail, Roche). For HDAC6/A3G-Vif interactions, cells were previously treated with the proteasome inhibitor ALLN (25 μM for 16 h), and then, when indicated, treated with RNAse A (1 mg/mL). After centrifugation, an input fraction (50 µL) was kept to check the protein expression level, and the rest was incubated for 2 h at 4°C with 2 µg of the different Abs (anti-HA, anti-EGFP, anti-HDAC6, anti-A3G or anti-Flag Abs) used to immunoprecipitate target proteins on a rotating wheel. After equilibration, protein G Dynabeads (Life Technologies) were added and incubated for 1.5 h at 4°C. Beads were washed five times with cold RIPA 1X buffer on a magnet, and eluted in NuPAGE LDS sample buffer 1X (Life Technologies) containing 50 mM glycine pH 2.8. After 10 min at 70°C, immunoprecipitated supernatants were loaded on NuPAGE 4–12% (Life Technologies) or SDS-PAGE 12% gradient gels, and analyzed together with the input cell fractions by western blot, as indicated above. To study the interaction of endogenous HDAC6 with endogenous A3G, CEM.NKR.CCR5 cells (5 × 10^6^) were used with the above experimental conditions. Moreover, when indicated, these cells were also nucleofected by pcDNA hVif vector (0.25 μg cDNA), to ascertain the existence of A3G/HDAC6-Vif ternary interactions.

### Purification and analysis of Vif-mediated ubiquitinated A3G

HEK 293T cells (2 × 10^6^) were transfected using PEI25k, as indicated above, with different plasmid combinations or by siRNA oligos. Thus, cells were transfected by using A3G-3xHA (2 μg), pcDNA-hVif (1 μg), HA-wt-HDAC6 (2 or 5 μg) or HA-HDAC6-ΔBUZ (2 or 5 μg) and Ubiquitin-6xHis (4 μg), which facilitates easy detection of Vif-mediated A3G ubiquitination. For RNA interference of HDAC6, cells were transfected with siRNA-HDAC6 oligos (1 μM) and Ubiquitin-6xHis (4 μg). pcDNA3.1 (2 μg) or scrambled oligos (1 μM) were used as control conditions. As Vif could be poly-ubiquitinated by itself, or by the same E3 ligase [34, 63], thereby entering into the proteasome degradation pathway [63, 93–95], 24 h after transfection, cells were treated with 20 μM of proteasome inhibitor MG132 for 5 h at 37°C and then washed three times in ice-cold PBS 1X, and lysed in 1 mL of Lysis Buffer (100 mM NaH_2_PO_4_ pH 8, 10% glycerol, 100 mM NaCl, 1 mM PMSF, 10 mM *N*-ethyl-maleimide, 0.2% Triton-X-100, 20 mM Imidazole in double-distilled water) for 30 min at 4°C. Lysates were centrifuged at 3,000×*g* for 15 min at 4°C and free-debris supernatants were collected. Protein analysis from inputs was carried out using these free-debris supernatants. Collected free-debris supernatants were next assayed to immobilize and purify ubiquitinated-6xHis proteins, including ubiquitinated overexpressed A3G-3xHA, by using Ni-NTA magnetic agarose beads (Qiagen, Turnberry Lane, Valencia, CA, USA) (40 μL of beads per 700 μL of total supernatant). Supernatants/Ni-NTA magnetic agarose bead mixes were incubated for 1 h at 4°C in a rotary shaker. Ni-NTA magnetic agarose beads, bearing attached ubiquitinated-6xHis-modified proteins, were precipitated and washed by using a magnet adapter [three times for 2 min each in 500 μL of washing buffer (100 mM NaH_2_PO_4_ pH 8, 300 mM NaCl, 0.2% Triton-X-100, 20 mM Imidazole in water)]. The remaining buffer was removed without disturbing the magnetic agarose beads. Ubiquitin-6xHis-protein complexes were eluted, and detached from their associated Ni-NTA magnetic agarose beads, in 50 μL of elution buffer (300 mM Imidazole in SDS-PAGE loading buffer 2X) at room temperature for 30 min. Eluted ubiquitinated proteins were analyzed by Western blot (SDS-PAGE 8%), as indicated above. In conjunction with an anti-HA mAb, they then were used to detect ubiquitinated A3G. By using an anti-HDAC6 rabbit polyAb, we detected HDAC6 constructs (HA-wt-HDAC6 and HA-HDAC6-ΔBUZ) co-immobilized and co-precipitated with ubiquitinated A3G-3xHA proteins. Non-ubiquitinated A3G-3xHA can also be precipitated by this method under any experimental condition. Using cell lysates (input), we analyzed (1) overexpressed HA-HDAC6 constructs with an anti-HDAC6 rabbit polyAb, which also permitted detection of endogenous HDAC6, (2) A3G-3xHA with an anti-HA mAb, (3) hVif with an anti-Vif mAb, and (4) α-tubulin with a specific mAb.

### Direct in vitro interaction assays

Full-length HA-tagged wt-HDAC6, HA-HDAC6-ΔBUZ and BUZ domain mutants, or HA-tagged HIV-1 Vif proteins were expressed in vitro using the rabbit-reticulocytes system, whereas GST, GST-A3G (wt or its mutants) and GST-Vif proteins were expressed in BL21-DE3 *E. coli* cells. HA-tagged proteins were resuspended in RIPA 1X buffer (PBS 1X, 1% NP40, 0.5% sodium deoxycholate, 0.05% SDS) supplemented with protease inhibitors (complete EDTA Free cocktail, Roche), and then purified using anti-HA Agarose beads following the manufacturer’s recommendations. For extraction of GST proteins, bacteria cells were lysed in Lysis Buffer (50 mM Tris–HCl pH 7.4, 2 mM EDTA, 150 mM NaCl, 1 mM DTT, 1% Triton X-100, 30 μg/mL Lysozyme, and protease inhibitors) and then purified by affinity using GSH-Sepharose beads. 2 μg of purified HA-tagged protein were incubated overnight in RIPA buffer at 4°C with 3 μg of GST, GST-A3G (wt, C97A or D128K mutants), or GST-Vif proteins immobilized on glutathione-Sepharose beads. Then, beads were washed three times in RIPA buffer and the bound cellular proteins were analyzed by Western blotting using the appropriate antibodies. Hence, in vitro expressed, purified and pulled-down HA-tagged wt, ΔBUZ or BUZ constructs of HDAC6, and HIV-1 Vif proteins were analyzed using an anti-HA monoclonal antibody. Loading of GST proteins was analyzed using an anti-GST antibody.

### Production of viral particles

Replication-deficient luciferase-HIV-1 viral particles were obtained as previously described [80–83, 92]; specifically, in HEK 293T-packaging cells that were also overexpressing different plasmid combinations of HA-HDAC6 constructs (2 μg) and/or A3G-3xHA (1 μg), or which had been treated with siRNA-HDAC6 (1–1.6 μM) or scrambles oligos (1–1.6 μM). Briefly, replication-deficient viral particles were derived by the luciferase-expressing reporter virus HIV/Δ*nef*/Δ*env*/*luc* + (bearing the luciferase gene inserted into the nef ORF and not expressing envelope glycoprotein) with an X4-tropic (Lai) or R5-tropic (SF162) envelope glycoprotein. X4- or R5-tropic HIV-1 viral particles were produced by co-transfecting HEK 293T cells (70% of confluence) in 10-cm^2^ dishes with pNL4-3.Luc.R-E- (10 μg) and X4-tropic (HXB2-env) or R5-tropic (pCAGGS SF162 gp160) Env glycoprotein (10 μg) vector and, when indicated, in cells expressing HA-HDAC6 constructs and/or A3G-3xHA, or treated with siRNA-HDAC6 or scrambled oligos. Viral plasmids were transduced in HEK 293T cells (12-well culture plates) using X-tremeGENE HP DNA transfection reagent as recommended by the manufacturer (Roche Diagnostics). After 6 h, medium was changed to RPMI 1640 and supplemented with 10% FCS and antibiotics, with the cells then cultivated for 48 h to allow viral production. Viruses were harvested 48 h post-transfection and HEK 293T cells were lysed to analyze the expression of Pr55^Gag^, proviral Vif, endogenous HDAC6 or overexpressed HA-HDAC6 proteins, A3G-3xHA, α-tubulin or actin. Supernatants containing cell-free viral particles were clarified by centrifugation at 3,000×*g* for 30 min, filtered by 0.45 μm, and concentrated by Amicon Ultra-4 Centrifugal filter devices (Millipore). Virions were used to infect HeLa P5 cells or were stored at −80°C. Viral stocks were normalized by p24-Gag content as measured with an enzyme-linked immunosorbent assay test (GenscreenTM HIV-1 Ag Assay) (Bio-Rad, Marnes-la-Coquette, France). To detect protein from nascent virions, supernatants from HEK 293T cells were centrifuged (3,000×*g* for 30 min), filtered (0.2 μm) and centrifuge for 4 h at 13,000×*g*. Then, total protein from higher density viral fraction was overnight precipitated by cold acetone (−20°C). Protein pellet was resuspended and treated by Laemmli buffer to detect proteins of interest (A3G, Vif and p24 on virions) by western blot, as above described.

### Luciferase viral entry and infection assay

HeLa-P5 cells (20,000 cells in 96-well plates with 20 μg/mL of Polybrene) were infected with a synchronous dose of luciferase-based X4- or R5-tropic HIV-1 viral inputs (500 ng of p24), in 200 μl RPMI 1640 medium for 5 h at 37°C. Virus was then removed by washing and subsequent trypsinization of infected cells. After 48 h of infection, luciferase activity (associated to viruses entry into infected cells) was determined from cell lysates by using a luciferase assay kit (Biotium, Hayward, CA, USA) with a microplate reader (VictorTM X5, PerkinElmer, Waltham, MA, USA), as described [80, 83]. Similarly, HIV-1 infection was measured by measuring β-galactosidase enzymatic activity in 48 h-infected cells (activity associated to cell lysates from infected HeL-P5 cells) using a β-Gal reporter gene assay (Roche Diagnostics), as previously described [44, 83]. Inhibition of luciferase or β-galactosidase activity was calculated for each dose point after subtracting background (in the presence of a neutralizing anti-CD4 mAb at 5 μg/mL), and the background of luciferase measurement/β-galactosidase activity in non-infected cells. Data were analyzed using GraphPad Prism 5.0 software (GraphPad Software, San Diego, CA, USA).

## Additional file

Additional file 1: Summary illustration. Model for HDAC6-mediated A3G protection and control of HIV-1 infectiveness. HDAC6 directly interacts and forms a constitutive complex with A3G. This HDAC6/A3G complex is formed either in the absence (**1**) or in the presence of the HIV-1 Vif protein (**2**), and appears to be independent on the BUZ domain of HDAC6 (the different domains of HDAC6 are represented). Moreover, HDAC6 could concomitantly interact with Vif (**3**). The balance between the level of HDAC6 and Vif expression conditions the efficiency of HDAC6 to interact with A3G (**1** and **2**) and/or Vif (**3** or **4**; in the absence or presence of A3G, respectively), to avoid A3G-Vif interaction and subsequent Vif-mediated A3G ubiquitination and proteasome degradation (promoting HIV-1 infection) (pathway **5**; the Vif-recruited E3 ligase complex (Vif-[CBF-β-EloB-EloC-Cul5-Rbx2/E2]) that targets A3G is shown). In fact, HDAC6 induces Vif autophagic clearance in a BUZ-dependent manner (pathway **3**), thereby inhibiting HIV-1 infectiveness. This event is dependent on the HDAC6-deacetylase activity and could be blocked by the 3-MA inhibitor (**6**), which perturbs membrane flux during autophagosome formation; a process promoted early on by HDAC6 during autophagy. Hence, HDAC6 competes for the Vif-A3G interaction and subsequent anti-A3G functions (avoiding pathway **5**). HDAC6 accounts for A3G steady-state expression level, stabilizing Vif-non-targeted forms of A3G (**1**). By these mechanisms, HDAC6 impairs HIV-1 infectiveness by Vif clearance (pathway **6**) and/or favouring A3G-mediated anti-HIV-1 functions (pathway **1**). Therefore, HDAC6/A3G appears as a new natural restriction complex acting against Vif function and HIV-1 infection.

## Abbreviations

HDAC6: histone deacetylase 6
BUZ: bound to ubiquitin zinc finger
A3G: apolipoprotein B mRNA-editing enzyme, catalytic polypeptide-like 3G (APOBEC3G)
CBF-β: core binding factor β
HIV: human immunodeficiency virus
Vif: viral infectivity factor
