# Supplementary figures and images for: The HDAC6/APOBEC3G complex regulates HIV-1 infectiveness by inducing Vif autophagic degradation
Source: Retrovirology. 2015 Jun 24;12:53. doi: 10.1186/s12977-015-0181-5 (PMC4479245; doi:10.1186/s12977-015-0181-5)

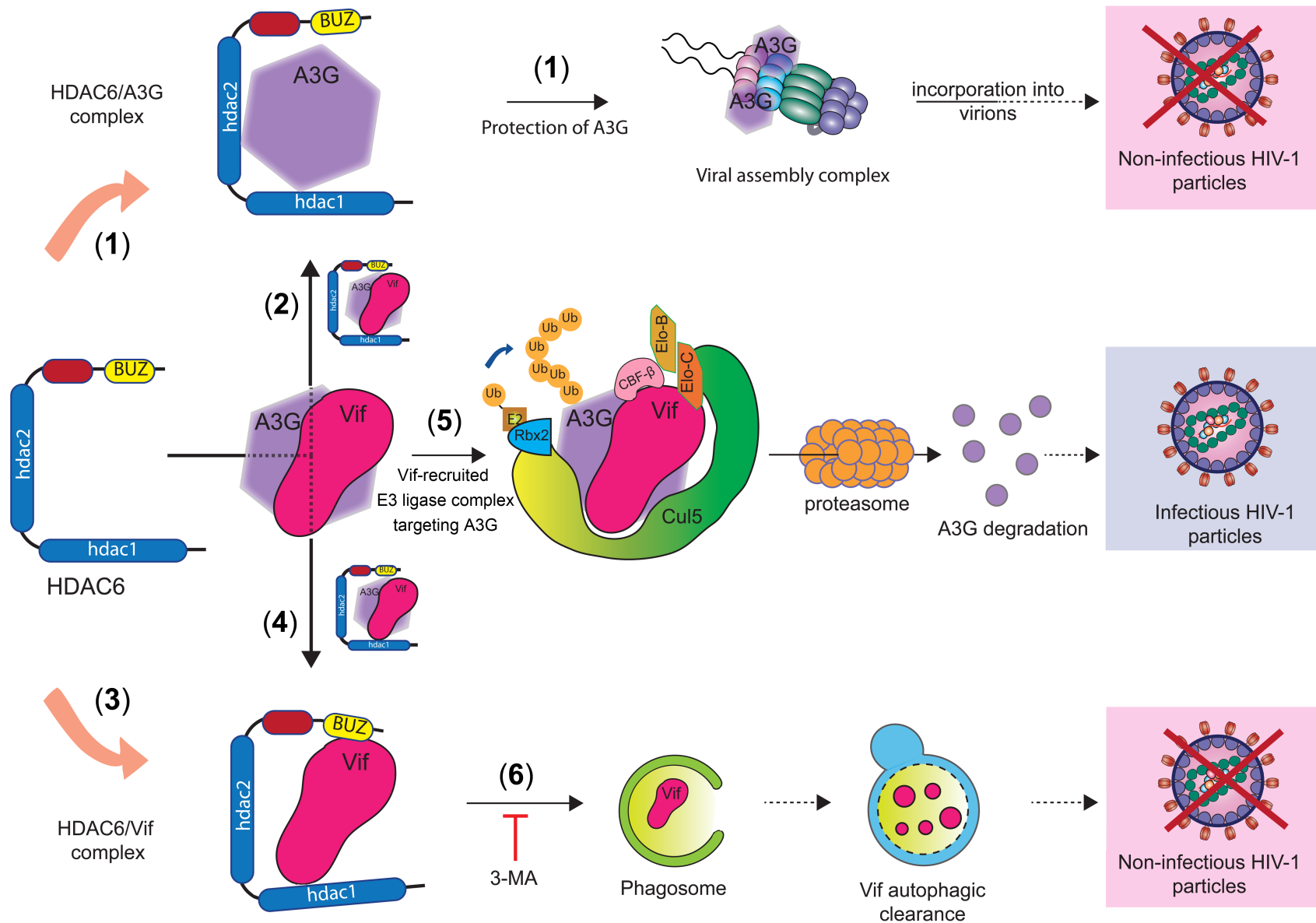

Supplement: Additional file 1: — Summary illustration. Model for HDAC6-mediated A3G protection and control of HIV-1 infectiveness. HDAC6 directly interacts and forms a constitutive complex with A3G. This HDAC6/A3G complex is formed either in the absence (1) or in the presence of the HIV-1 Vif protein (2), and appears to be independent on the BUZ domain of HDAC6 (the different domains of HDAC6 are represented). Moreover, HDAC6 could concomitantly interact with Vif (3). The balance between the level of HDAC6 and Vif expression conditions the efficiency of HDAC6 to interact with A3G (1 and 2) and/or Vif (3 or 4; in the absence or presence of A3G, respectively), to avoid A3G-Vif interaction and subsequent Vif-mediated A3G ubiquitination and proteasome degradation (promoting HIV-1 infection) (pathway 5; the Vif-recruited E3 ligase complex (Vif-[CBF-β-EloB-EloC-Cul5-Rbx2/E2]) that targets A3G is shown). In fact, HDAC6 induces Vif autophagic clearance in a BUZ-dependent manner (pathway 3), thereby inhibiting HIV-1 infectiveness. This event is dependent on the HDAC6-deacetylase activity and could be blocked by the 3-MA inhibitor (6), which perturbs membrane flux during autophagosome formation; a process promoted early on by HDAC6 during autophagy. Hence, HDAC6 competes for the Vif-A3G interaction and subsequent anti-A3G functions (avoiding pathway 5). HDAC6 accounts for A3G steady-state expression level, stabilizing Vif-non-targeted forms of A3G (1). By these mechanisms, HDAC6 impairs HIV-1 infectiveness by Vif clearance (pathway 6) and/or favouring A3G-mediated anti-HIV-1 functions (pathway 1). Therefore, HDAC6/A3G appears as a new natural restriction complex acting against Vif function and HIV-1 infection. [file 12977_2015_181_MOESM1_ESM.pdf]
